# MRE11 deacetylation by SIRT2 promotes DNA binding to facilitate DNA end resection and ATM-dependent signaling

**DOI:** 10.1172/JCI186711

**Published:** 2026-01-08

**Authors:** Fatmata Sesay, Hui Zhang, Priya Kapoor-Vazirani, Andrew T. Jung, Mark E. Essien, Amanda J. Bastien, Nho C. Luong, Xu Liu, PamelaSara E. Head, Duc M. Duong, Xiaofeng Yang, Zachary S. Buchwald, Xingming Deng, Nicholas T. Seyfried, David S. Yu

**Affiliations:** 1 Department of Radiation Oncology and Winship Cancer Institute, Emory University School of Medicine, Atlanta, Georgia, USA.; 2 Department of Biology, Clark Atlanta University, Atlanta, Georgia, USA.; 3 Department of Biochemistry, Emory University School of Medicine, Atlanta, Georgia, USA.

**Keywords:** Cell biology, Oncology, DNA repair, Radiation therapy

## Abstract

MRE11, a breast tumor suppressor and component of the MRE11-RAD50-NBS1 complex, plays a critical role in DNA end resection and initiation of ataxia-telangiectasia mutation–dependent (ATM-dependent) DNA damage signaling. However, the precise mechanisms governing MRE11 function in the DNA damage response (DDR) remain incompletely understood. Here, we found that MRE11 is deacetylated by the SIRT2 sirtuin deacetylase and breast tumor suppressor, which promotes DNA binding to facilitate DNA end resection and ATM-dependent signaling. SIRT2 deacetylase activity promoted DNA end resection. SIRT2 further complexed with and deacetylated MRE11 at conserved lysine 393 (K393) in response to DNA double-stranded breaks (DSBs), which promoted MRE11 localization and DNA binding at DSBs but not interaction with RAD50, NBS1, or CtIP. Moreover, MRE11 K393 deacetylation by SIRT2 promoted ATM-dependent signaling. Our findings define a mechanism regulating MRE11 binding to DNA through SIRT2 deacetylation, elucidating a critical upstream signaling event directing MRE11 function in the DDR and providing insight into how *SIRT2* dysregulation leads to genomic instability and tumorigenesis.

## Abstract 2

We define a role for the SIRT2 sirtuin deacetylase and breast tumor suppressor in directing MRE11 function in promoting DNA end resection and ATM-dependent DNA damage signaling by facilitating its binding to DNA through deacetylation

## Graphical abstract

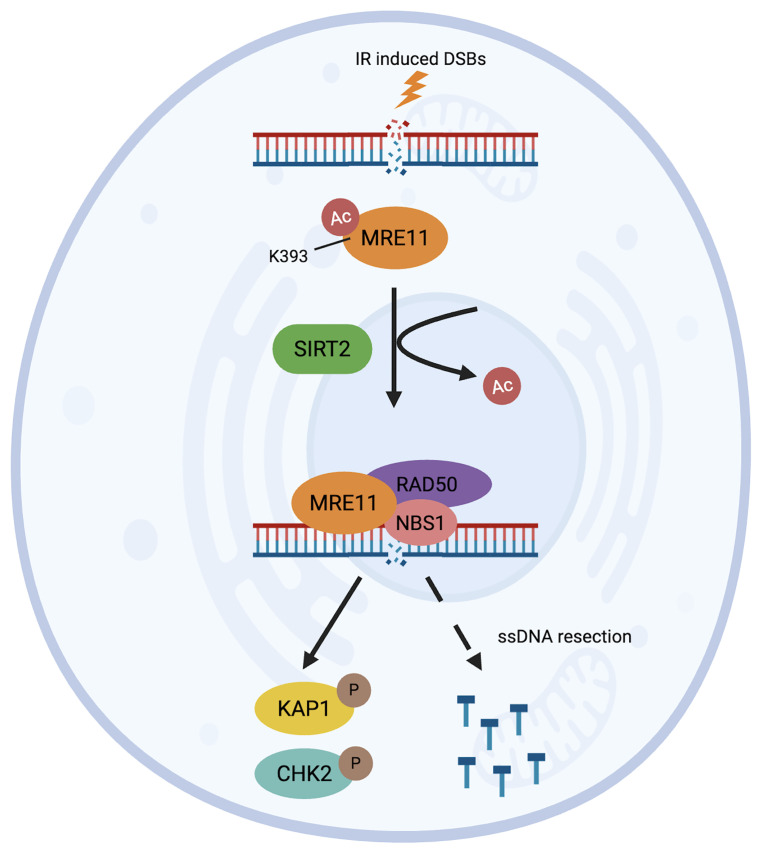

## Introduction

DNA double-stranded breaks (DSBs) are the most deleterious type of DNA damage and must be repaired to preserve genome integrity (1–3). DSB repair defects can lead to multiple disease types, including cancer, aging, neurological dysfunction, immune deficiency, and multiple human syndromes (4, 5). Meiotic recombination 11 (MRE11), a component of the MRE11-RAD50-NBS1 complex, plays a critical role in responding to DSBs through pleiotropic functions, including sensing, processing, and signaling, which activates the DNA damage response (DDR) (6–8). MRE11 recognizes, binds, and bridges DSBs (9–12). MRE11 also processes DSB ends through both exonuclease and endonuclease activities to generate short, 3′ ssDNA overhangs (13–17), which are critical for homologous recombination–mediated (HR-mediated) DSB repair (18). Furthermore, MRE11 is involved in the activation of the ataxia-telangiectasia mutated (ATM) checkpoint kinase (19–21), a central regulator of the DDR. Upon induction of DSBs, ATM is recruited and phosphorylates downstream targets involved in cell cycle checkpoints, DNA repair, and apoptosis, including CHK2 and KAP1 (22–26). The multifaceted functions of MRE11 in DSB sensing, DNA end resection, and ATM-dependent signaling underscore its critical role orchestrating cellular responses to DNA damage and maintaining genome integrity. Significantly, Mre11 suppresses murine breast tumorigenesis and metastasis (27, 28), and dysregulated *MRE11* expression and/or mutations are found in a number of cancer types, including germline mutations in breast cancer (29–37). Moreover, *MRE11* is mutated in individuals with ataxia-telangiectasia–like disorder (38–45), providing further evidence that MRE11 dysregulation contributes to human disease.

MRE11 binds to ssDNA and dsDNA directly through both amino-terminal and carboxyl terminal DNA-binding domains (9, 12, 46–50). This interaction is also facilitated by, as well as indirectly mediated by, RAD50 (49, 51, 52) and NBS1 (48, 53, 54), which are critical for MRE11 activities in the DDR. However, the mechanisms regulating MRE11 recruitment and binding to DNA are not well understood. MRE11 has been reported to be modified by lactylation (55) and methylation (56–59), which promote DNA binding, and phosphorylation, which dissociates it from chromatin (60–63). However, a role for acetylation in directing MRE11’s functions in the DDR remains unclear.

Sirtuin 2 (SIRT2), an NAD^+^-dependent deacetylase, is recognized for its role in diverse biological processes, including genome maintenance, tumor suppression, aging, and metabolism (64–67). Studies involving mice lacking *Sirt2* have demonstrated an increased susceptibility to breast, liver, and other cancer types, suggesting a role for SIRT2 in tumor suppression (68, 69). Moreover, somatic cancer-associated *SIRT2* mutations impair SIRT2’s activity in genome maintenance (70), suggesting a role for SIRT2 in tumor suppression that may be attributed, at least in part, to a role in genome maintenance. Indeed, SIRT2 acts as a central regulator of DDR pathways by modulating the acetylation status of several proteins involved in the DDR, including ATRIP (71), BARD1 (72), RAD52 (73), CDK9 (74), and DNA-PKcs (75). However, a role for SIRT2, and more generally of sirtuins, in regulating MRE11 function as well as a role for SIRT2 in directing DNA end resection and ATM checkpoint signaling, which are facilitated by MRE11, is not known.

Here, we show that MRE11 deacetylation at conserved lysine 393 (K393) by SIRT2 promotes its localization and binding to DNA at DSBs to promote DNA end resection and ATM-dependent signaling. Our findings define a mechanism governing MRE11 function in the DDR, elucidating a critical upstream signaling event directing MRE11’s role in DNA end resection and ATM-dependent signaling via SIRT2 deacetylation.

## Results

### SIRT2 interacts with and deacetylates MRE11 in response to DSBs.

**Figure 1:**
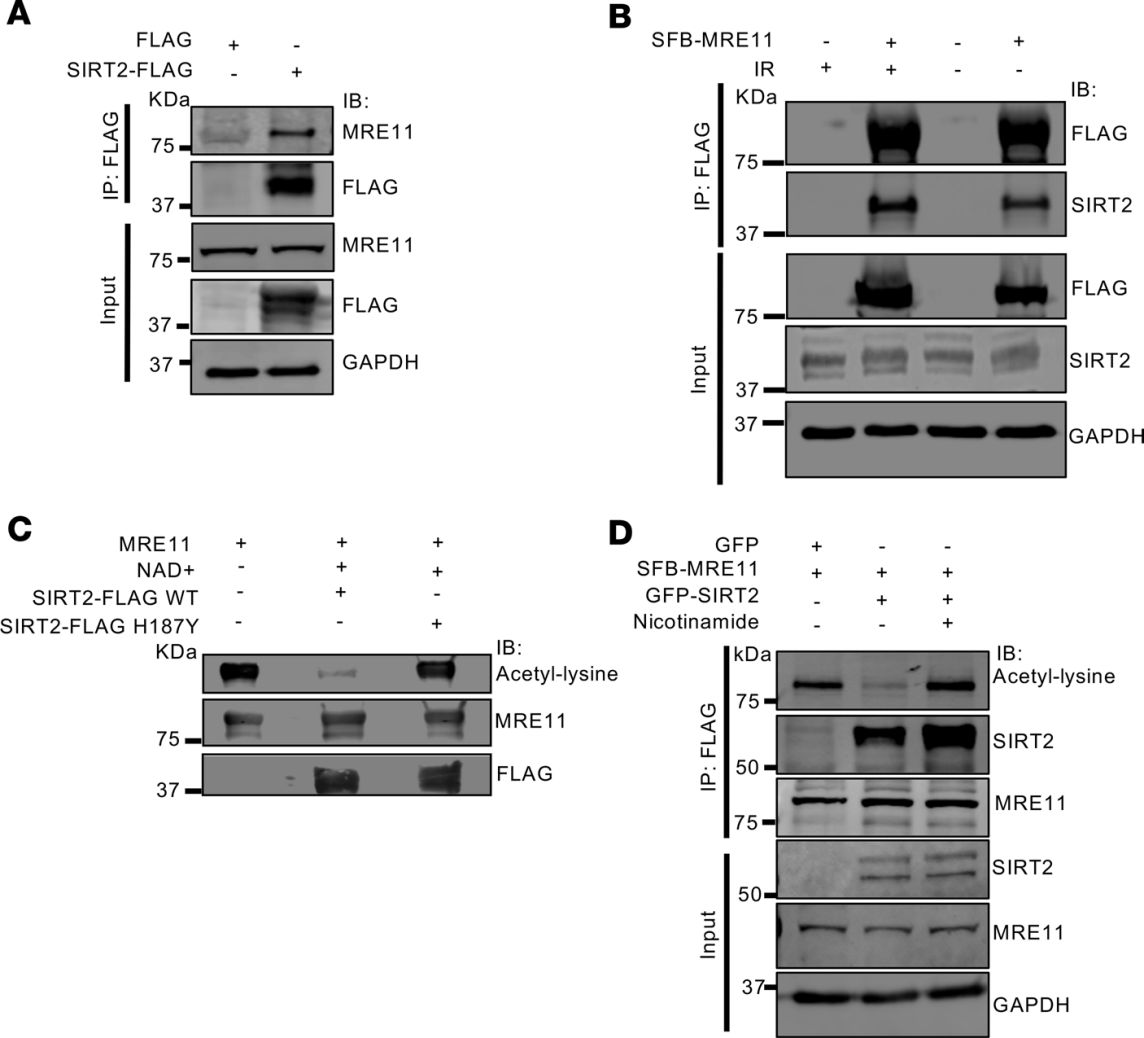
SIRT2 complexes with and deacetylates MRE11. (**A**) IP of SIRT2 with anti-FLAG antibody from HEK293T cells expressing SIRT2-FLAG or FLAG empty vector, followed by Western blot with the indicated antibodies. (**B**) Reciprocal co-IP of MRE11 from HEK293T cells expressing SFB-MRE11 or empty vector with and without 6 Gy IR and a 4-hour recovery period. (**C**) In vitro deacetylation assay of MRE11 by SIRT2. Immunopurified acetylated GFP-MRE11 was incubated with immunopurified SIRT2-FLAG WT or catalytically inactive H187Y in an in vitro reaction with and without NAD. (**D**) Cellular deacetylation assay of MRE11 by SIRT2. HCT116 cells expressing SFB-MRE11 and GFP-SIRT2, with or without nicotinamide, were harvested, immunoprecipitated with anti-FLAG antibody, and subjected to SDS-PAGE and Western blot with the indicated antibodies. Shown are representative Western blots; each experiment was conducted 3 independent times.

To investigate the interaction between SIRT2 and MRE11, we conducted co-immunoprecipitation (co-IP) analyses. We expressed SIRT2-FLAG in human embryonic kidney (HEK) 293T cells and observed its interaction with endogenous MRE11 (Figure 1A). In reciprocal co-IP experiments, SFB-MRE11 expressed in HEK 293T cells pulled down endogenous SIRT2 (Figure 1B). We found no evidence for a change in interaction of MRE11 with SIRT2 in response to DNA damage induced by 6 Gy ionizing radiation (IR) (Figure 1B), consistent with SIRT2’s interaction with several DDR substrates (71, 75). To assess the deacetylation of MRE11 by SIRT2, we conducted both in vitro and cellular deacetylation assays. SIRT2-FLAG WT, but not deacetylase inactive SIRT2-FLAG H187Y (76), deacetylated purified acetylated GFP-MRE11 in vitro (Figure 1C). Furthermore, GFP-SIRT2 deacetylated SFB-MRE11 expressed in HCT116 cells in a manner that was inhibited by nicotinamide, a sirtuin inhibitor (Figure 1D). Collectively, these data indicate SIRT2 complexes with and deacetylates MRE11 in vitro and in cells.

### SIRT2 promotes MRE11 localization to DNA damage sites.

**Figure 2:**
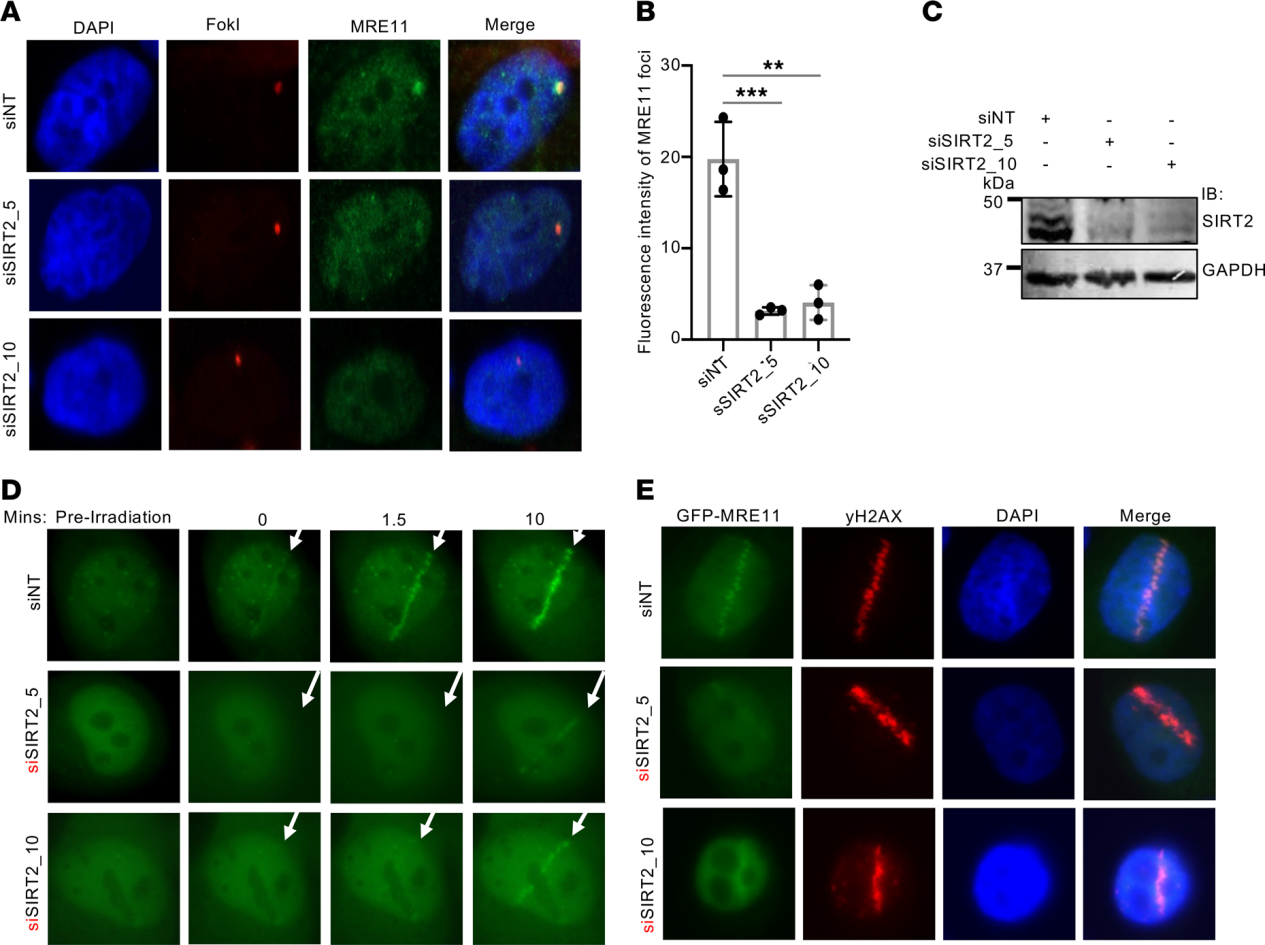
SIRT2 promotes MRE11 localization to DNA damage sites. (**A** and **B**) U2OS-265 cells were transfected with SIRT2 or nontargeting (NT) siRNA and induced for FokI expression. FokI and MRE11 proteins were visualized by mCherry fluorescence and anti-MRE11 antibody. DNA was visualized by DAPI staining. Representative confocal microscopy images (**A**) and quantification of MRE11 fluorescence intensity at the FokI-damage site (**B**) are shown. Data are reported as the mean ± SD from 3 replicates of 50 cells. (**C**) Western blot showing SIRT2 depletion in U2OS-265 cells used in **A** and **B**. (**D** and **E**) MRE11 recruitment to DNA damage sites induced by laser-microirradiation is impaired after SIRT2 depletion. (**D**) U2OS cells with SIRT2 or NT siRNA and transfected with GFP-MRE11 were subjected to laser microirradiation. A representative image shows the time course of GFP-MRE11 accumulation at laser-induced damage sites (arrows). (**E**) U2OS cells were transfected with GFP-MRE11 and SIRT2 or NT siRNA, and then subject to laser microirradiation, followed by IF for γH2AX to verify DNA damage. The representative image shows accumulation of GFP-MRE11 and γH2AX at sites of laser-induced damage. All representative IF experiments were conducted at least 3 independent times. The Western blots shown are representative, and each experiment was conducted at least 3 independent times. Statistical significance was determined using 1-way ANOVA followed by Dunnett’s post hoc test for multiple comparisons. **P < 0.01 and ***P < 0.001.

To determine the functional significance of the interaction of SIRT2 and MRE11, we assessed the impact of SIRT2 on MRE11’s localization to DNA damage sites, using U2OS-265 cells, in which 4-hydroxytamoxifen (4-OHT) triggers nuclear translocation of FokI nuclease domain fused to mCherry-LacR (mCherry-LacR-FokI) to induce DSBs at a chromosomally integrated 256-copy lac operator array (77). SIRT2 depletion impaired the localization of endogenous MRE11 to FokI-induced DSBs in these cells (Figure 2, A–C). To validate our observation, we induced DNA damage in U2OS cells through microirradiation with a 365 nm laser. Notably, SIRT2 depletion impaired the mobilization of GFP-MRE11 to DNA damage sites induced by laser microirradiation co-labeled with γH2AX, a marker for DSBs (Figure 2, D and E), suggesting that SIRT2 promotes MRE11 localization to DSBs.

### SIRT2 deacetylase activity promotes DNA end resection.

**Figure 3:**
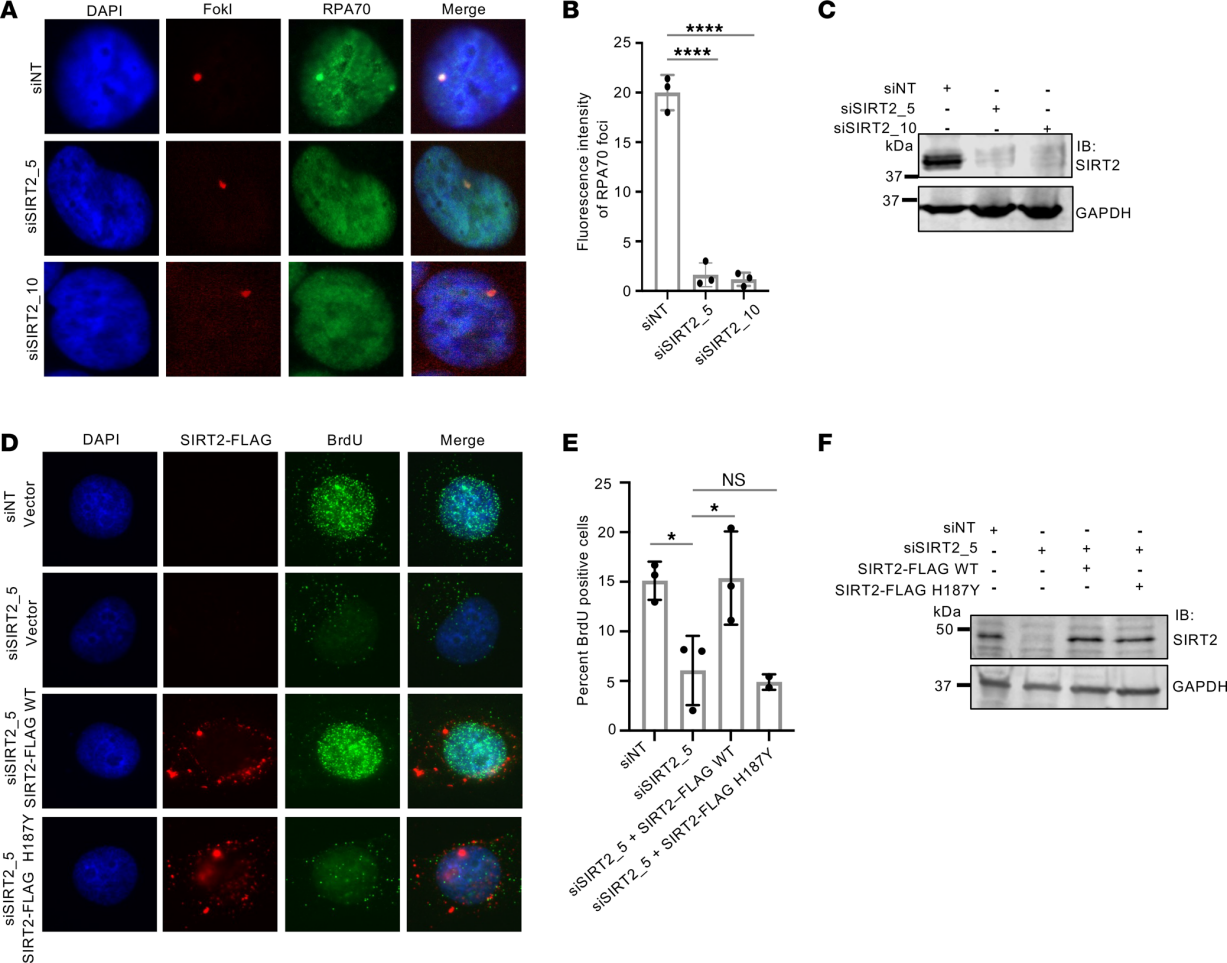
SIRT2 deacetylase activity promotes DNA end resection. (**A**–**C**) U20S-265 cells were treated with SIRT2 or NT siRNA and induced for FokI expression with 1 μM Shield-1 and 2 μM 4-OHT for 4 hours. Localization of FokI and endogenous RPA70 proteins were visualized by mCherry fluorescence and anti-RPA70 antibody. DNA was visualized by DAPI staining. (**A**) Representative images obtained by confocal microscopy are shown. (**B**) Quantitation of RPA70 fluorescence intensity at FokI-induced DSB site. Data are reported as the mean ± SD of 3 replicates of 50 cells. (**C**) Western blot showing knockdown of SIRT2 in the U2OS-265 cells used in **A** and **B**. (**D** and **E**) HeLa cells depleted for SIRT2 and rescued with siRNA-resistant SIRT2 WT or SIRT2 H187Y. Cells were processed for BrdU exposure. Representative images (**D**) and quantitation of relative percent BrdU foci positive cells (**E**) are shown. Data in the graph represent the mean ± SD. (**F**) Western blot analysis showing confirmation of SIRT2 depletion and expression of SIRT2-FLAG WT and H187Y. The Western blots shown are representative, and each experiment was conducted at least 3 independent times. Statistical significance was determined using 1-way ANOVA followed by Dunnett’s post hoc test for multiple comparisons. *P < 0.05 and ****P < 0.0001.

Given SIRT2’s role in directing MRE11 localization to DSBs, we investigated its involvement in the resection of DNA ends at DSBs. SIRT2 depletion in U2OS-265 cells impaired the localization of RPA70, which binds to resected ssDNA, to FokI-induced DSBs (Figure 3, A–C) and at DNA damage sites induced by laser microirradiation (Supplemental Figure 1; supplemental material available online with this article; https://doi.org/10.1172/JCI186711DS1). To more directly determine the role of SIRT2 in promoting DNA end resection, we examined BrdU exposure under nondenaturing conditions following treatment of HeLa cells with 6 Gy IR. Similar to CtIP but in contrast to 53BP1, SIRT2 depletion significantly impaired BrdU exposure but not γH2AX foci under these conditions (Supplemental Figure 2, A–C). Moreover, the impairment in BrdU exposure was rescued by the expression of SIRT2-FLAG WT but not H187Y (Figure 3, D–F). Collectively, these data suggest that SIRT2 deacetylase activity promotes DNA end resection in response to DSBs and that this is generalizable to multiple cell types.

### MRE11 K393 deacetylation by SIRT2 in response to DNA damage promotes its binding to dsDNA.

**Figure 4:**
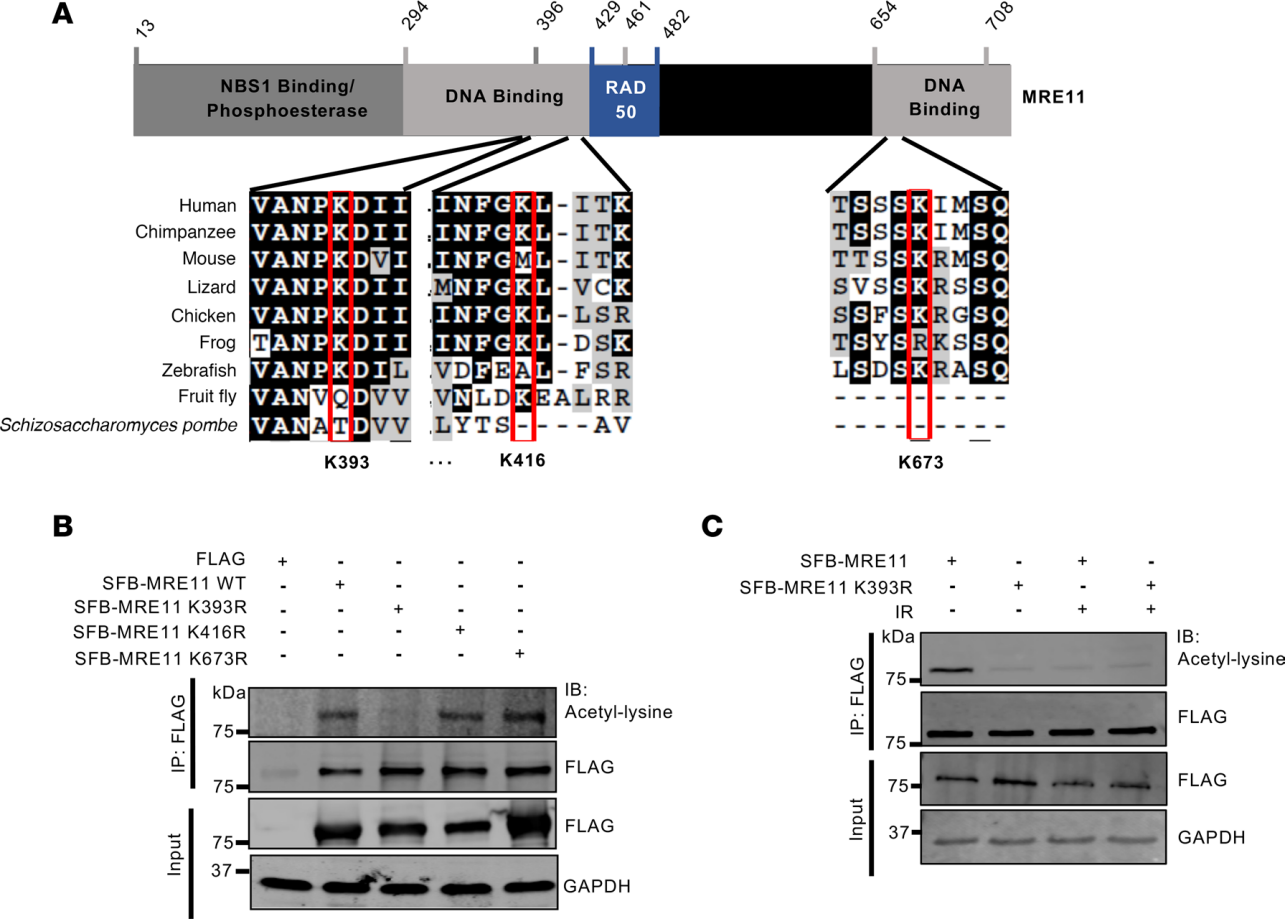
MRE11 is acetylated at K393. (**A**) Schematic of MRE11 protein depicting key domains and evolutionary conservation of putative Lys acetylation sites identified by liquid chromatography-MS/MS. NBS1 binding, RAD50 binding, and DNA binding domains are indicated. (**B**) Analysis of MRE11 site-specific Lys acetylation was tested with SFB-MRE11 WT and Lys to arginine nonacetylable Lys-mimic mutants expressed in HEK293T cells. Lysate from cells was immunoprecipitated with anti-FLAG, run on SDS-PAGE, and probed with indicated antibodies. (**C**) MRE11 K393 was deacetylated in response to IR. Cells expressing SFB-MRE11 WT and K393R were treated with or without 6 Gy IR, harvested after 4 hours, run on SDS-PAGE, immunoprecipitated with anti-FLAG, and probed with indicated antibodies. Western blots shown are representative, and each experiment was conducted at least 3 independent times.

To identify the specific lysine (Lys) residue (hereafter identified by K and the residue number**)** target sites of SIRT2 deacetylation on MRE11, we conducted mass spectrometry (MS) analysis of purified SFB-MRE11 expressed in HEK293T cells. Our analysis revealed 3 candidate acetylation sites on MRE11: K393, K416, and K673 (Figure 4A and Supplemental Figure 3A). Notably, among these sites, K393 and its surrounding amino acids located within MRE11’s more amino-terminal DNA binding domain showed the most evolutionary conservation (Figure 4A). Moreover, in silico bioinformatics analyses using Sorting Intolerant from Tolerant (SIFT) (78, 79) and PolyPhen-2 (80) indicated that mutations at K393 are likely to be the most impactful among these sites (Supplemental Figure 3B).

To identify Lys sites on MRE11 that undergo acetylation, we generated SFB-MRE11 mutants in which a single Lys residue was replaced by arginine, mimicking a nonacetylatable Lys. These mutants were then expressed in HEK293T cells and subjected to treatment with and without IR. In contrast to SFB-MRE11 K416R and K673R, which had comparable levels of acetylation to SFB-MRE11 WT at baseline, SFB-MRE11 K393R had reduced acetylation levels (Figure 4B), suggesting that K393 contributes to MRE11 acetylation. Moreover, IR induced a decrease in acetylation specifically in SFB-MRE11 WT, but not in the K393R mutant (Figure 4C), suggesting that MRE11 K393 is deacetylated in response to IR. Biochemical fractionation studies further showed that, like total endogenous MRE11, acetylated endogenous MRE11 is primarily nuclear and deacetylated in response to IR (Supplemental Figure 3C).

**Figure 5:**
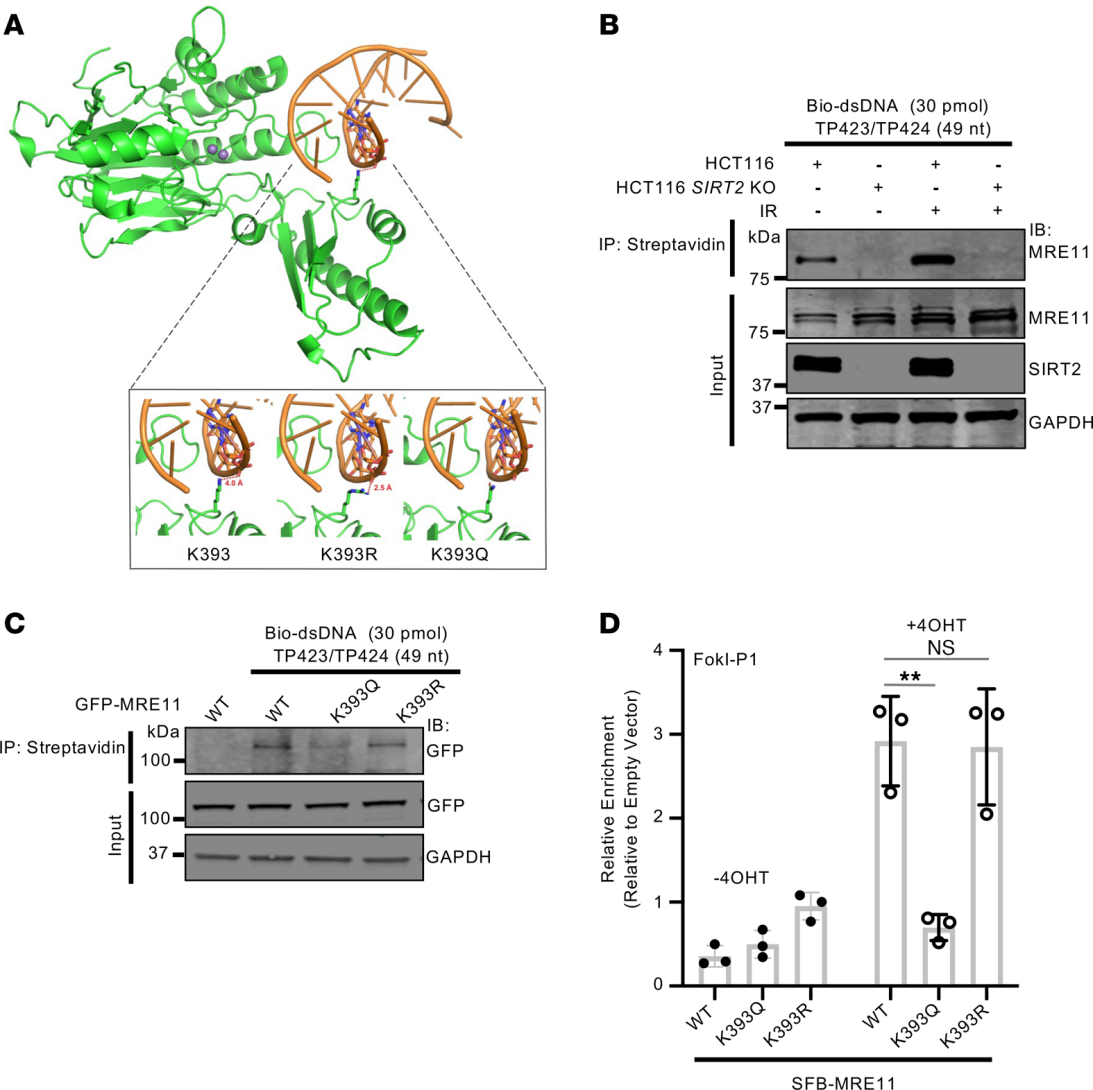
MRE11 K393 deacetylation by SIRT2 in response to DNA damage promotes its binding to dsDNA. (**A**) Structural model depicting the impact of K393 acetylation on binding with DNA. Human MRE11 aa 1–411 (PDB: 3T1I) were aligned with the structure of *Pyrococcus furiosus* MRE11-DNA complex (PDB: 3DSC). The distance between K393 and the phosphate group in the DNA was measured using PyMOL (version 2.5.4). Residue K393 was mutated to Q and R, respectively, in PyMOL. (**B**) MRE11 was purified from HCT116 SIRT2 WT and KO cells treated with or without 6 Gy IR with a 4 hour recovery, incubated with biotin-labeled dsDNA oligos (bio-dsDNA), and pulled down with agarose-streptavidin beads. Pull-downs were separated by SDS-PAGE and immunoblotted with the indicated antibodies. (**C**) GFP-MRE11 WT, K393Q, and K393R mutants were purified from HCT116 cells and incubated with bio-dsDNA as described in **B**. (**D**) Quantitative analysis of MRE11-binding to FokI-induced DSBs using ChIP-qPCR. Quantification from 3 independent experiments is shown as the mean ± SD. Western blots shown are representative, and each experiment was conducted at least 3 independent times. Statistical significance was determined using 1-way ANOVA followed by Dunnett’s post hoc test for multiple comparisons. **P < 0.01.

The location of K393 within MRE11’s DNA binding domain and impairment of MRE11 localization to DSBs after SIRT2 depletion suggests a critical role for K393 in mediating DNA binding. We aligned the structure of human MRE11 residues 1–411 (81) with that of *Pyrococcus furiosus* MRE11-DNA complex (12) and found potential electrostatic interaction between the positively charged K393 side chain (-NH_3_^+^) and the negatively charged DNA phosphate backbone (within 4.0 Å). This interaction may be impaired by acetylation of K393, as shown by the model of the K393Q mutation (Figure 5A). To determine whether MRE11 K393 deacetylation by SIRT2 is critical for its interaction with DNA, we conducted streptavidin pulldown assays using biotin-labeled dsDNA incubated with MRE11 purified from HCT116 *SIRT2* WT and KO cells treated with or without IR. An increase in interaction of MRE11 with dsDNA was observed after IR treatment, and the interaction was impaired with *SIRT2* deficiency (Figure 5B). Moreover, both GFP-MRE11 WT and K393R had increased binding to dsDNA compared with GFP-MRE11 K393Q, which mimics an acetylated state (Figure 5C). This trend was consistent in A549 IR-resistant cells, where GFP-MRE11 WT and K393R also exhibited increased binding to dsDNA relative to GFP-MRE11 K393Q, and IR treatment did not alter DNA binding, consistent with the IR-resistant phenotype (Supplemental Figure 3D).

To determine whether K393 acetylation impacts MRE11 binding to DSBs, we performed ChIP quantitative PCR (qPCR) of SFB-MRE11 WT, K393Q, and K393R expressed in U2OS-265 cells. SFB-MRE11 WT and K393R, but not K393Q, showed increased binding to DSBs after 4-OHT treatment (Figure 5D and Supplemental Figure 3, E and F). In contrast, we observed no substantial difference in interaction of SFB-MRE11 WT, K393R, and K393Q with RAD50, NBS1, and CtIP by co-IP (Supplemental Figure 3G), which suggests K393 acetylation does not affect its interactions with RAD50, NBS1, and CtIP and likely does not disrupt global MRE11 protein structure. Collectively, these findings indicate SIRT2 deacetylation of MRE11 at K393 facilitates its binding to dsDNA at DSBs.

### MRE11 K393 deacetylation by SIRT2 promotes DNA end resection and HR.

**Figure 6:**
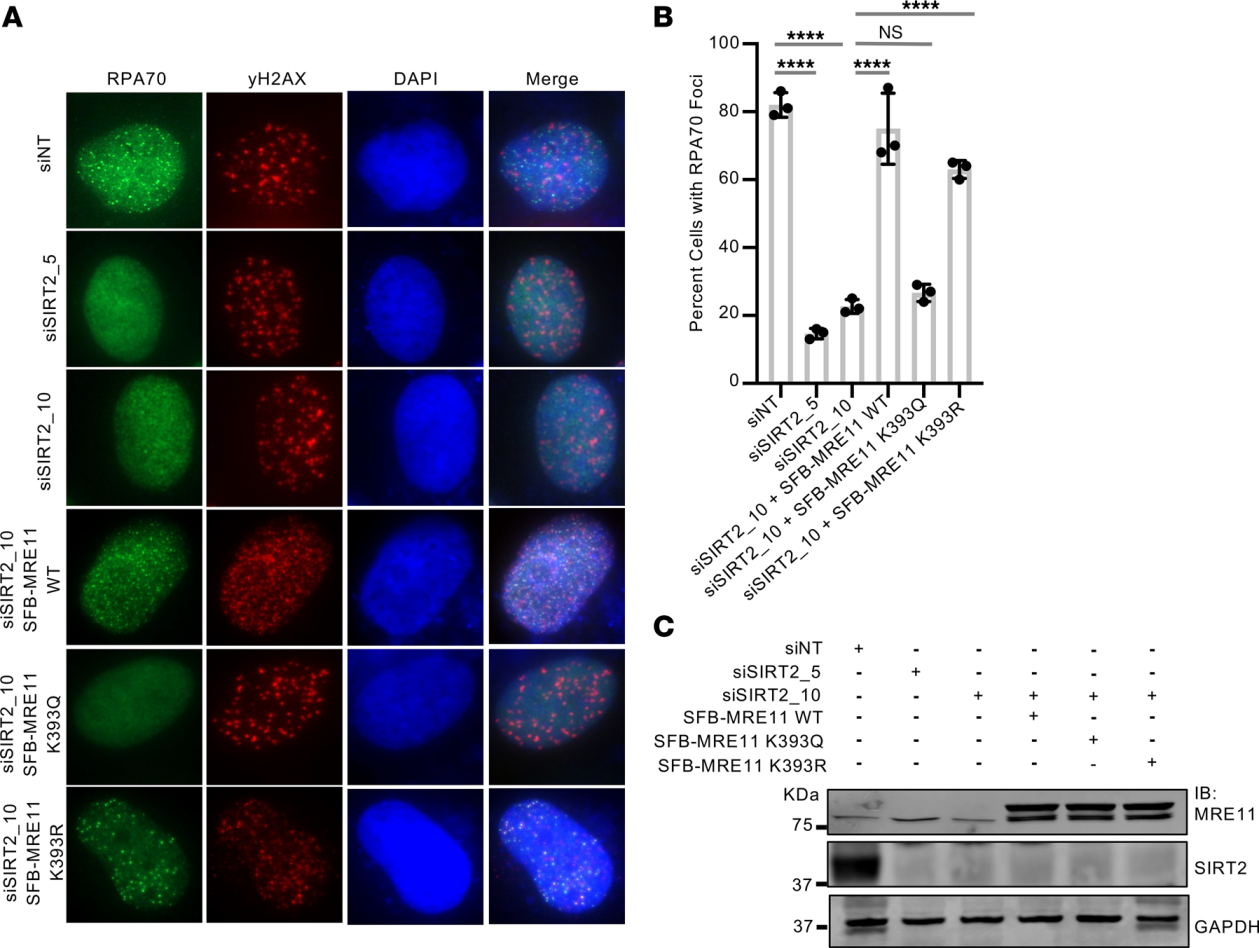
MRE11 K393 deacetylation by SIRT2 promotes DNA end resection. (**A**) U20S cells, either not silenced or silenced for SIRT2 and expressing SFB-MRE11 proteins where indicated, were treated with 6 Gy IR and recovered for 4 hours. Cells were processed for IF with antibodies against RPA70 and γH2AX. Shown is a representative image. (**B**) Percentage of cells with RPA70 foci (per positive γH2AX foci cell) in **A** was quantified across 3 independent experiments. Data are reported as the mean ±- SD. (**C**) Western blot analysis showing SIRT2 silencing and SFB-MRE11 expression in **A**. Western blots shown are representative and each experiment was conducted at least 3 independent times. Statistical significance was determined using 1-way ANOVA followed by Dunnett’s post hoc test for multiple comparisons. ****P < 0.0001.

**Figure 7:**
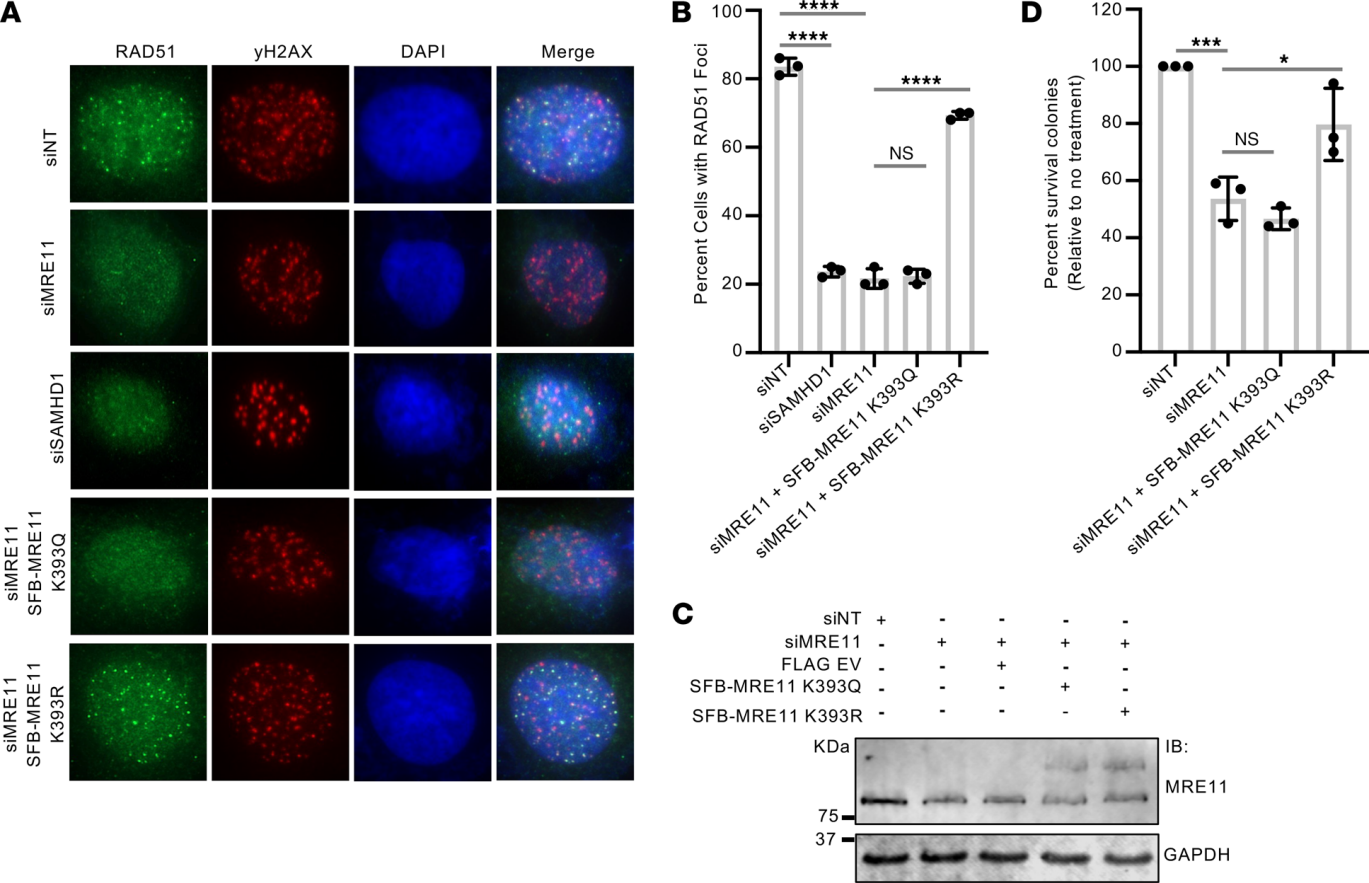
MRE11 acetylation at K393 impairs HR. (**A**) U20S cells were either not silenced or silenced for MRE11 and expressing SFB-MRE11 proteins where indicated, then treated with 6 Gy IR and recovered for 4 hours. Cells were processed for IF with antibodies against RAD51 and γH2AX. (**B**) The percentage of cells with RAD51 foci was calculated. Three independent experiments were conducted, and data are presented as the mean ± SD. (**C**) Western blot indicating downregulation of MRE11 and expression of SFB-MRE11 in **A**. (**D**) Colony formation assay in U2OS cells after MRE11 knockdown and re-expression of mutant MRE11 proteins. Percentages of colonies surviving relative to no treatment (siNT) are shown. The experiment was conducted 3 independent times, and data are represented as the mean ± SD. Western blots shown are representative, and each experiment was conducted at least 3 independent times. Statistical significance was determined using 1-way ANOVA followed by Dunnett’s post-hoc test for multiple comparisons. *P < 0.05, *** *P* < 0.001, and ****P < 0.0001. NT, nontargeting.

To determine the downstream functional significance of MRE1 K393 deacetylation by SIRT2, we examined IR-induced RPA70 foci in U2OS cells silenced for SIRT2 and expressing SFB-MRE11 WT, K393R, and K393Q. The impairment in IR-induced RPA70 foci following SIRT2 depletion was alleviated by expression of SFB-MRE11 WT and K393R, but not K393Q (Figure 6, A–C), suggesting that MRE11 K393 deacetylation by SIRT2 promotes DNA end resection. Moreover, expression of SFB-MRE11 K393R, but not K393Q, alleviated the impairment in IR-induced RAD51 foci and IR hypersensitivity after MRE11 depletion in U2OS cells (Figure 7A-D), suggesting that MRE11 deacetylation promotes HR and IR resistance.

### MRE11 K393 deacetylation by SIRT2 promotes ATM-dependent signaling.

**Figure 8:**
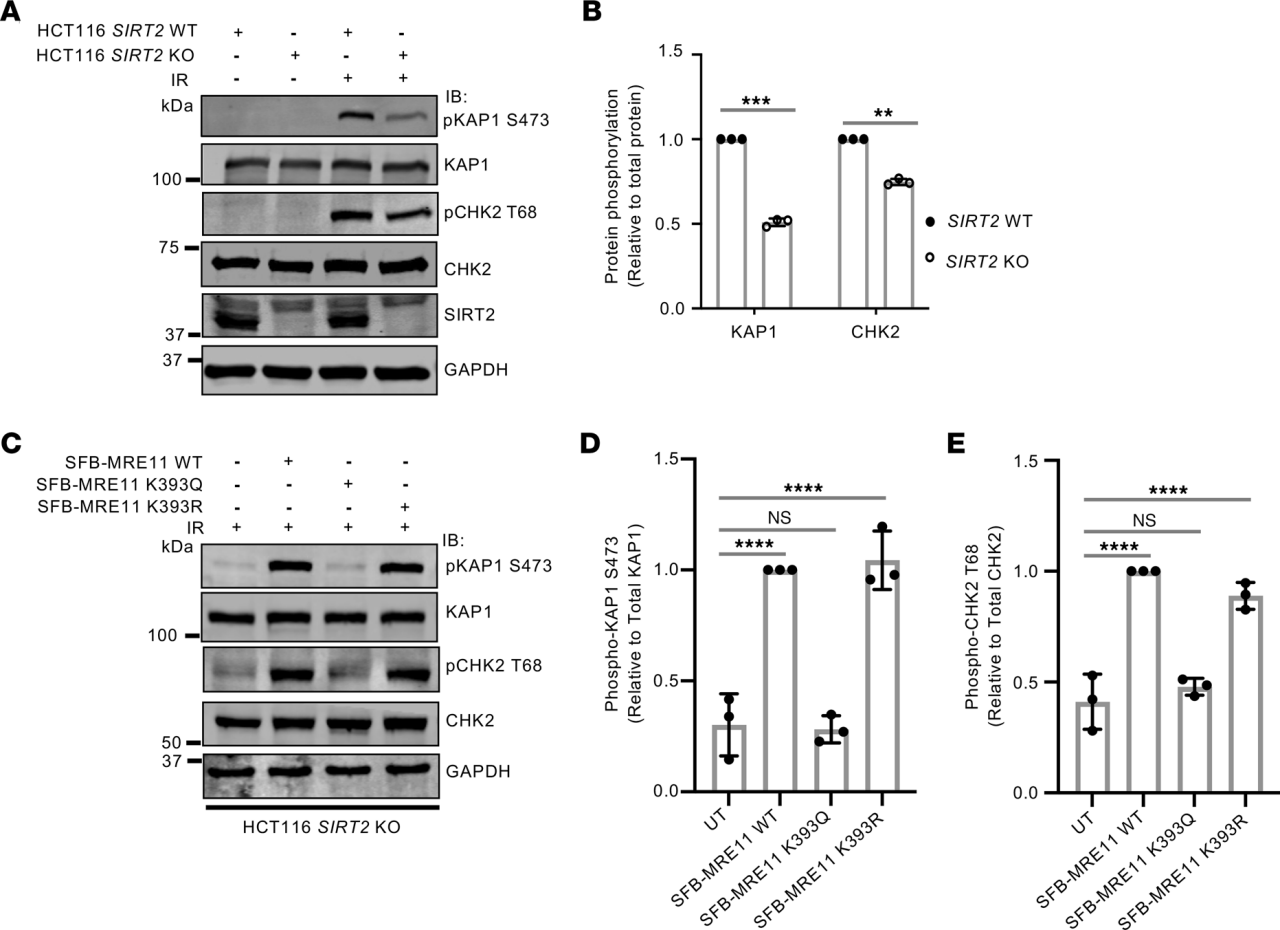
MRE11 K393 deacetylation by SIRT2 promotes ATM-dependent signaling. (**A**) HCT116 SIRT2 WT and KO cells were treated with 6 Gy IR, harvested after 4 hours, run on SDS-PAGE, and probed with the indicated antibodies. The image shown is representative of data from 3 separate experiments conducted independently. (**B**) Quantitation of IR-induced phosphorylation of KAP1 and CHK2 relative to total KAP1 and CHK2 protein levels. Data are represented as the mean ± SD from 3 independent experiments. **P < 0.01 and ***P < 0.001, by 2-tail Student’s *t* test. (**C**) HCT116 *SIRT2* KO cells expressing SFB-MRE11 WT, K393Q, and K393R were treated with or without 6 Gy IR, harvested after 4 hours, run on SDS-PAGE, and probed with the indicated antibodies. Shown is a representative image of 3 independent experiments. (**D** and **E**) Quantitation of phosphorylation of KAP1 and CHK2 relative to total KAP1 and CHK2 protein levels. Data are presented as the mean ± SD of 3 independent experiments. Statistical significance was determined using 1-way ANOVA followed by Dunnett’s post hoc test for multiple comparisons. ****P < 0.0001.

To determine if K393 deacetylation by SIRT2 affects ATM-dependent signaling, we examined ATM-dependent phosphorylation of KAP1 at serine 473 (S473) and CHK2 at threonine 69 (T69) in HCT116 *SIRT2* WT and KO cells after treatment of the cells with 6 Gy IR, which activates the DDR in these cells (75). The IR-induced ATM-dependent phosphorylation of KAP1 at S473 and CHK2 at T69 but not total KAP1 or CHK2 levels were impaired with SIRT2 deficiency (Figure 8, A and B), suggesting that SIRT2 promotes ATM signaling. Significantly, the impairment in ATM-dependent phosphorylation of KAP1 at S473 and CHK2 at T69 in HCT116 S*IRT2* KO cells was rescued by expression of SFB-MRE11 WT and K393R, but not by K393Q expression (Figure 8, C–E), suggesting that MRE11 K393 deacetylation by SIRT2 promotes ATM-dependent signaling. However, we found no evidence that *SIRT2* deficiency in HCT116 cells or SFB-MRE11 K393Q expression in MRE11-silenced IR-resistant A549 cells (IRR-A549) impairs ATM-dependent phosphorylation of NBS1 at S343 (82–84) (Supplemental Figure 4, A and B), which is activated by SIRT1 deacetylation of NBS1 (85), thus suggesting that MRE11 K393 deacetylation by SIRT2 does not promote ATM-dependent phosphorylation of all ATM substrates.

## Discussion

Our findings reveal a critical regulatory mechanism governing MRE11 function in the DDR, whereby MRE11 deacetylation at conserved K393 by SIRT2 facilitates MRE11 localization and binding to DNA at DSBs, thereby promoting DNA end resection and ATM-dependent DNA damage signaling. These findings reveal a critical upstream signaling event directing MRE11 localization to DNA damage sites and binding to DNA that promotes MRE11’s functions in the DDR, identify MRE11 as a unique binding partner and substrate for SIRT2, and establish SIRT2 as a positive regulator of DNA end resection and ATM-dependent signaling. Moreover, our findings provide insight into how *SIRT2* deficiency results in genomic instability and development of cancer. In this regard, we found that SIRT2 deacetylase activity promotes DNA end resection, defining a role for SIRT2 in DNA end resection that facilitates its known function in DSB repair by HR (72, 73, 86) and governing resistance to IR (75, 87). We further found that SIRT2 complexes with and deacetylates MRE11 at conserved K393 located in a region critical for interfacing with DNA (12), providing evidence that MRE11 is an interacting partner and substrate for SIRT2. In addition, we found that K393 deacetylation by SIRT2 promotes MRE11 localization and binding to DNA at DSBs, providing a mechanistic model for how SIRT2 promotes MRE11 function in the DDR. Finally, we found that K393 deacetylation by SIRT2 promotes DNA end resection, HR, IR resistance, and ATM-dependent signaling, demonstrating functional significance of K393 deacetylation by SIRT2 in promoting MRE11 downstream functions in the DDR.

**Figure 9:**
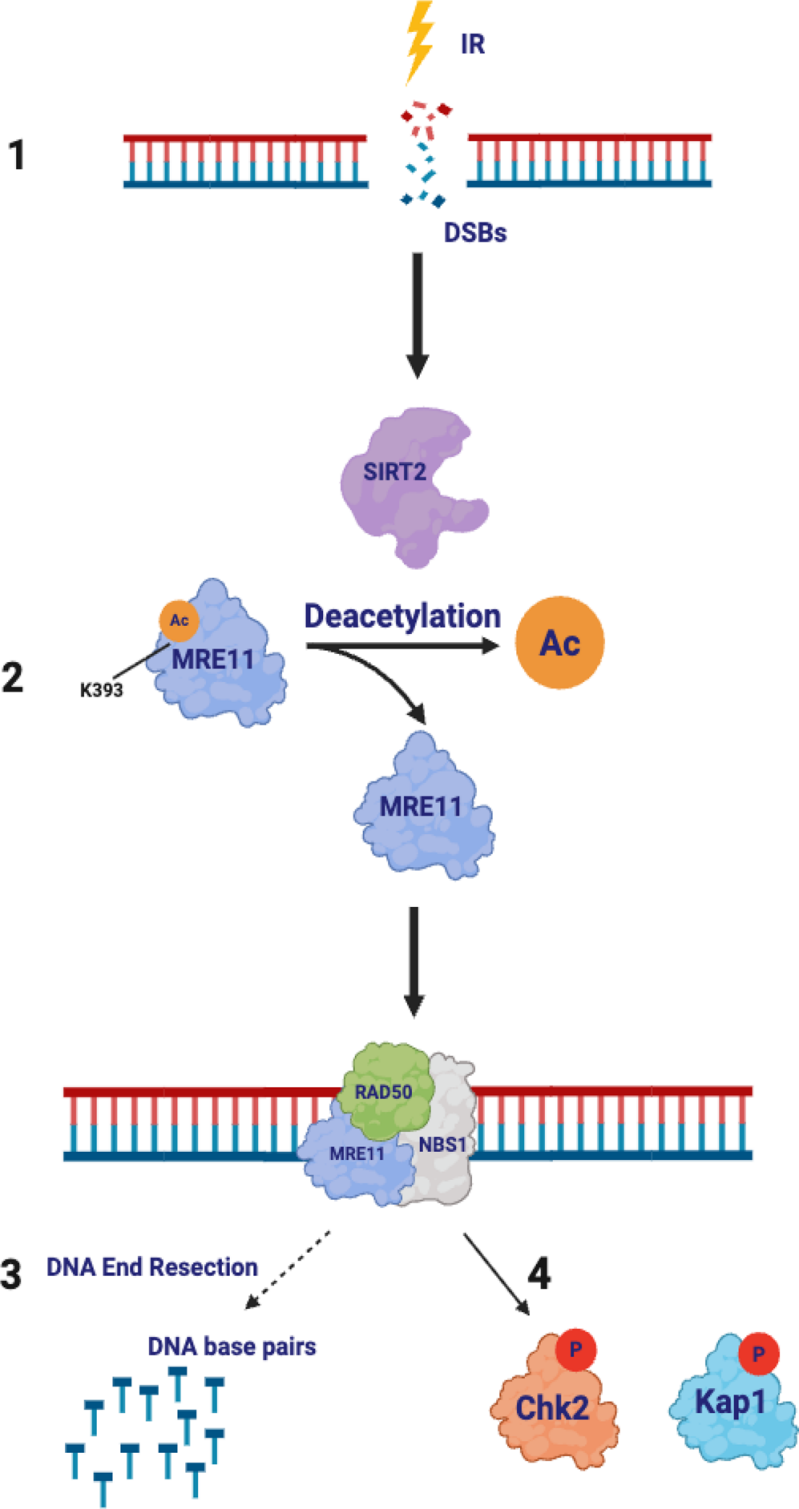
Model of SIRT2 regulation of MRE11 in the DDR. (Step 1) DNA damage results in DSBs. (Step 2) SIRT2 deacetylates MRE11 at K393 in response to DNA damage. (Step 3) MRE11 K393 deacetylation by SIRT2 facilitates its recruitment and binding to DNA at DSBs. (Step 4) MRE11-RAD50-NBS1 assembles and promotes DNA end resection and ATM-dependent phosphorylation of downstream substrates, including CHK2 and KAP1.

Our findings thus lead to a model in which, after IR, SIRT2 deacetylates MRE11 at K393 to facilitate its localization and binding to dsDNA at DSBs, which promotes DNA end resection and ATM-dependent phosphorylation of downstream substrates, including CHK2 and KAP1 (Figure 9). Dysregulation of this pathway leads to genomic instability, which may contribute to SIRT2’s role in tumor suppression.

The amino and carboxyl-terminal domains of MRE11 bind to DNA (9, 12, 46–50), which is facilitated by RAD50 and NBS1 (49, 51, 52). Our observation that SIRT2 deficiency impairs MRE11 binding to dsDNA and that MRE11 K393Q, but not WT or K393R, has impaired binding to dsDNA and FokI-induced DSBs suggests a critical role for SIRT2 in directing reversible acetylation at K393 within the amino-terminal DNA binding domain to facilitate MRE11 binding to dsDNA. At a structural level, our modeling indicates that the positive charge of K393 mediates electrostatic interactions with the negative charge of nucleic acids interfacing with MRE11, and these are disrupted by acetylation. Our finding that K393 acetylation does not impair MRE11 interaction with RAD50, NBS1, or CtIP suggests that K393 does not mediate binding with DNA indirectly through these proteins. Furthermore, we found no evidence that K393 acetylation impairs NBS1 S343 phosphorylation under the same conditions of impairment in DNA binding, suggesting that the effect of acetylation status of MRE11 on DNA binding is distinct from that of the acetylation status of NBS1 on NBS1 S343 phosphorylation, which is regulated by SIRT1 (85). MRE11 is modified by lactylation at K673 (55) and by methylation at R570 and R594 within its glycine-arginine–rich motif (55), which promote DNA binding, and by phosphorylation at S649, 676, and 678, which dissociates it from chromatin (55). We now report a critical role for acetylation at K393 within MRE11’s amino-terminal DNA binding domain in regulating its binding with DNA, which complements regulation at its carboxyl-terminal DNA domain. Given that K393 acetylation alone abolishes most of MRE11’s binding with DNA, these data suggest a central role for K393 acetylation in governing MRE11 binding with DNA that cannot be fully compensated for by its carboxyl-terminal DNA-binding interface.

Our data indicate that MRE11 K393 is deacetylated by SIRT2 in response to IR; however, we found no evidence that the interaction of SIRT2 and MRE11 is regulated by IR. It is possible that IR-regulated deacetylation of MRE11 results from an increase in SIRT2 catalytic activity induced by IR. Indeed, we previously reported that the SIRT2 deacetylase activity increases in response to DNA damage in vitro (71). SIRT2 directs HR through deacetylation of BARD1 (72) and recruitment of RPA70 and RAD51 to DSBs through an undefined mechanism (73). Our observation that SIRT2 promotes DNA end resection provides evidence for SIRT2 in directing an early event that facilitates HR. Moreover, our finding that SIRT2 promotes ATM-dependent signaling through MRE11 deacetylation broadens SIRT2’s role in the ATM checkpoint pathway in addition to directing the ATR (71) and DNA-PKcs (75) PI3K-related kinases, and provides further support for SIRT2 in regulating a network of proteins involved in the DDR (67). In addition, our finding that SIRT2 directs MRE11 in DNA end resection and ATM-dependent signaling provides additional insight into how both *SIRT2* and *MRE11* dysregulation leads to genomic instability and breast tumorigenesis.

## Methods

### Sex as a biological variant.

Both male and female patients’ cancer cell lines were used in this study; however, we do not expect sex differences to contribute to the presented phenotypes.

### Inclusion and diversity.

In curating references pertinent to our research, we made a concerted effort to ensure gender balance within our References. Additionally, our article’s authorship comprises individuals from the research locale who were actively involved in data collection, design, analysis, and/or interpretation of the findings.

### Cell lines.

Human HEK293T*,* HeLa*,* HCT116 *SIRT2* WT and *KO*, and U2OS cells, all from ATCC, were cultured in DMEM (Gibco, Thermo Fisher Scientific) supplemented with 7.5% FBS. U2OS-265 mCherry-LacI-FokI cells, provided by Roger Greenberg (University of Pennsylvania, Philadelphia, Pennsylvania, USA), were cultured in DMEM supplemented with 10% FBS, 2 μg/mL puromycin, and 100 μg/mL hygromycin. IRR-A549 cells were cultured in DMEM/F12 medium supplemented with 10% FBS. All cell lines were incubated under humidified conditions at 37°C with 5% CO_2_ and 95% air.

### Transfections.

The siRNA duplexes were obtained from Dharmacon or Qiagen. Plasmid DNA and siRNA transfections were performed using BioT (Bioland Scientific) and RNAi Max transfection reagent (Invitrogen, Thermo Fisher Scientific), respectively, following the manufacturer’s instructions. Individual siRNAs were purchased from Dharmacon or Qiagen. These sequences were as follows: NT(ATGAACGTGAATTGCTCAATT); SIRT2-5(GGAGAAAGCTGGCCAGTCG); SIRT2-10 (TGGGCAGAAGACATTGCTTAT); CtIP (GCUAAAACAGGAACGAAUC); and 53BP1 (GAAGGACGGAGUACUAAUAUU).

### Immunoblot.

Cells were harvested in PBS and lysed for 30 minutes on ice in NP40 buffer (200 mM NaCl, 1% NP40, 50 mM Tris-HCl, pH 8.0) freshly supplemented with protease inhibitors (Thermo Fisher Scientific, 78438). After lysis, the samples were centrifuged at 15,682 × *g* for 10 minutes at 4°C, and the resulting supernatants were collected. Protein concentrations were determined using the Bradford assay. Subsequently, protein samples were resolved by SDS-PAGE, transferred onto PVDF membranes, and probed with the appropriate primary antibodies. Detection was performed using the LI-COR Odyssey system. The antibodies used in this study included SIRT2 (MilliporeSigma, 09-843; Santa Cruz Biotechnology, sc-20966), GAPDH (Santa Cruz, sc-47724), Flag (Santa Cruz Biotechnology, sc-51590), GFP (Abcam, Ab6556), pan acetyl-Lys (Santa Cruz Biotechnology, sc-8649), RPA70 (Cell Signaling Technology, 2267), P-CHK2 T68 (Cell Signaling Technology, 2661S), CHK2 (Santa Cruz Biotechnology, sc-7898), P-KAP1 (Invitrogen, Thermo Fisher Scientific, MA1-2023), and KAP1 (Bethyl Laboratories, A300-767A).

### Immunoprecipitation.

Briefly, cells were harvested and washed once with PBS. Cells were then lysed for 30 minutes on ice with CHAPS buffer (10% [vol/vol] glycerol, 150 mM NaCl, 50 mM Tris pH 7.5.75% CHAPS) with the usual protease inhibitors added fresh. When probing for acetylation, 1 μM trichostatin A (TSA) and 20 μM nicotinamide were added with the protease inhibitors. The cells were then centrifuged at 15,682 × *g* for 15 minutes at 4°C and the resulting pellet discarded. An equal volume of minus CHAPS buffer (10% [vol/vol] glycerol, 150 mM NaCl, 50 mM Tris pH 7.5) was added to the supernatant to dilute the CHAPS concentration to 0.375%. The lysate was precleared via incubation for 1 hour with 30 μL of either protein G agarose beads (Invitrogen, Thermo Fisher Scientific) or protein A agarose beads (Invitrogen, Thermo Fisher Scientific). Protein G agarose beads were used when the IP antibody was mouse; protein A agarose beads were used when the IP antibody was rabbit. The lysate was then added to 30 μL of preconjugated beads overnight on a rotator at 4°C. FLAG-tagged proteins were immunoprecipitated using FLAG M2 affinity beads (MilliporeSigma); GFP-tagged proteins were immunoprecipitated using GFP antibody (Abcam, Ab6556). Endogenous IP was done with the indicated antibody. Negative controls consisted of IP with lysate using IgG rabbit or mouse for endogenous IP and lysate not expressing tagged proteins for nonendogenous IP. The beads were then washed 3 times with the 0.375% CHAPS buffer and then resuspended in 15 μL of 0.375% CHAPS buffer and 5 μL of 4× Laemmli sample buffer before being boiled for 5 minutes at 100°C. They then underwent SDS-PAGE.

### IF analysis.

U20S or U2OS-265 mCherry-LacI-FokI cells were transfected with NT, SIRT2, or MRE11 siRNA, as well as SFB-MRE11 plasmids where indicated. After transfection, U20S cells were treated with 6 Gy IR and grown for 4 hours. U2OS-265 mCherry-LacI-FokI cells were treated with 1 μM Shield-1 (TaKaRa, 632189) and 2 μM 4-hydroxytamoxifen (4-OHT) (MilliporeSigma, H7904-5mg) for 4 hours. Cells were fixed with 3% paraformaldehyde for 10 minutes at room temperature, permeabilized with 0.5% Triton X-100 for 10 minutes, and then blocked with 5% BSA in PBS. Immunostaining was performed using anti-MRE11 (GeneTex, GTX70212), anti-RPA70 (MilliporeSigma, 2267), anti-RAD51 (Cell Signaling Technology, 14223), and anti-γH2AX (MilliporeSigma, 05-636) antibodies, followed by Alexa Fluor 488 or 555 secondary antibodies (Invitrogen, Thermo Fisher Scientific, A11034 and A21424). DNA was visualized with DAPI (Southern Biotech, 0100-20). Images were acquired using a Zeiss Observer Z1 microscope equipped with Axiovision Rel 4.8 software, with samples scanned using a ×60 oil objective. The fluorescence intensity of MRE11 or RPA70 foci was determined using ImageJ software.

### BrdU foci DNA end resection assay.

HeLa cells were transfected with NT, SIRT2, CtIP, or 53BP1 siRNA, as well as SIRT2-FLAG plasmids where required. Cells were grown for 48 hours after plasmid transfection and then incubated with 30 μM BrdU for 36 hours, followed by further incubation for an additional 4 hours with 6 Gy IR or 5 μM camptothecin, a topoisomerase I inhibitor, which induces single-ended DSBs that are repaired primarily by HR. Cells were pre-extracted with extraction buffer 1 (10 mM PIPES at pH 7.0, 300 mM sucrose, 100 mM NaCl, 3 mM MgCl_2_, 1 mM EGTA, and 0.5% Triton X-100), washed with PBS, incubated in extraction buffer 2 (10 mM Tris-HCl at pH 7.5, 10 mM NaCl, 3 mM MgCl_2_, 1% Tween 40, and 0.5% sodium deoxycholate) and further washed with PBS. Cells were subsequently fixed and permeabilized for IF, as described in the previous subsection. Primary antibodies used were against BrdU (BD Biosciences, 347580), γH2AX (Cell Signaling Technology, 9718), and FLAG (Cell Signaling Technology, 2368).

### In vitro deacetylation assay.

Experiments were performed as previously described (88, 89). Briefly, 293T cells were transiently transfected with plasmids encoding GFP-tagged MRE11 and histone acetyltransferases (p300/CBP and pCAF). Prior to harvesting, cells were treated overnight with 0.5 μM TSA and 20 mM nicotinamide. Harvested cells were lysed using IP buffer (20 mM HEPES at pH 7.4, 180 mM KCl, 0.2 mM EGTA, 1.5 mM MgCl_2_, 20% glycerol, 1.0% Nonidet P-40) supplemented with 1 μM TSA. GFP-MRE11 proteins were immunoprecipitated using anti-GFP antibody. Immunoprecipitants were washed with IP buffer containing 1 μM TSA to remove nicotinamide and prepared for in vitro deacetylation assay. Agarose beads with conjugated GFP-MRE11 (300 ng) were then suspended in 25 μL of deacetylation reaction buffer (50 mM Tris-HCl, pH 7.5, 150 mM NaCl, 1 mM MgCl_2_) with or without 10 mM NAD and supplemented with purified SIRT2 (1 μg) at 30 °C for 3 hours with constant agitation. For purification of SIRT2, Flag-SIRT2 (WT or H187Y) was transiently transfected into 293T cells. After protein expression, cells were lysed using IP buffer. The protein lysates were immunoprecipitated using anti-FLAG M2 affinity beads (MilliporeSigma). Immunoprecipitants were washed with IP buffer followed by TBS (150 mM NaCl, 50 mM Tris, pH 7.5). Immunoprecipitated proteins were eluted with TBS supplemented with 0.15 mg/mL 3×FLAG peptide (MilliporeSigma) and prepared for in vitro deacetylation assay. The reaction was terminated by the addition of 5× SDS-loading buffer. Samples were then analyzed for acetylation levels by Western blot using an anti–pan acetyl Lys antibody (Santa Cruz Biotechnology, sc-8649).

### Cellular deacetylation assay.

For cellular deacetylation analysis, HCT116 cells were transiently transfected with SFB-MRE11 with or without GFP-SIRT2-WT, followed by treatment with or without nicotinamide (an SIRT2 inhibitor). The cells were cultured with 0.5 μM TSA for 12 hours. Subsequently, the cells were lysed using IP buffer supplemented with 1 μM TSA. Protein lysates were then immunoprecipitated using anti-FLAG M2 affinity beads (MilliporeSigma, F2426). The immunocaptured proteins were subjected to deacetylation analysis by immunoblotting using an anti-acetyl antibody (Santa Cruz Biotechnology, sc-8649).

### Biotinylated DNA pull-down assay.

HCT116, HCT116 *SIRT2* KO, and IR-resistant A549 cells, expressing GFP-MRE11 plasmids where required, were treated or not treated with 6 Gy IR and grown for 4 hours. Cells were lysed on ice for 20 minutes in lysis buffer containing 10 mM Tris-HCl at pH 7.5, 100 mM NaCl, 10% glycerol, 10 μg/mL BSA, 0.05% NP40, CHAPS 0.35%, and protease inhibitors. Whole-cell lysates (600 μg) were precleared for 1 hour at 4°C with 30 μL of washed streptavidin-conjugated agarose beads (MilliporeSigma, 69203). Precleared lysates were incubated with 30 μL of streptavidin-conjugated beads only or beads prebound to 30 pmol of biotinylated dsDNA overnight at 4°C. Samples were washed with lysis buffer 3 times and boiled in 5× SDS sample buffer; Western blot with MRE11 (GeneTex, GTX70212) or GFP (Abcam, ab290) antibodies then was performed. Western blotting also was conducted of 50 μg of input lysate with GFP, MRE11, SIRT2 (MilliporeSigma, 09-8430), GAPDH (Santa Cruz Biotechnology, sc-47724), and Tubulin (MilliporeSigma, T6074) antibodies.

### ChIP assay.

ChIP was performed using the ChromaFlash One-Step ChIP Kit (P-2025, Epigentek) following the manufacturer’s protocol. Briefly, Fok1 cells were transfected with the SFB-MRE11 WT and mutant constructs for 72 hours and treated with 1 μM Shield-1 and 4-OHT for 4 hours to induce damage. The cells were then fixed with 1% formaldehyde (MilliporeSigma, 252549) for 15 minutes at room temperature to crosslink DNA-protein interactions. Excessive crosslinking was quenched by adding 0.125 M glycine for 5 minutes. Cells were washed 3 times with ice-cold PBS for 5 minutes each time, and cell lysates were prepared using 0.375% CHAPS lysis buffer (10% glycerol, 150 mM NaCl, 50 mM Tris at pH 7.5). Chromatin was fragmented into an average size of 500–800 base pairs using a Branson microtip SFX250 sonicator. IP was performed using Flag antibody, as described earlier in Methods. Flag empty vector was used as an internal control. After IP, chromatin was subjected to DNA isolation, and real-time qPCR was performed using PowerTrack SYBR Green Master Mix A46012 on a PCR 7500 Fast Real-Time PCR system (Thermo Fisher Scientific). Primers specific for various DNA regions, including FokI-P1, FokI-P2, and FokI-P3 prone repair sites in FokI cells, were used. Primer sequences were as follows: Fok1-P1: forward – GGAAGATGTCCCTTGTATCACCAT, reverse – TGGTTGTCAACAGAGTAGAAAGTGAA; Fok1-P2: forward – GCTGGTGTGGCCAATGC, reverse – TGGCAGAGGGAAAAAGATCTCA; and Fok1-P3: forward - GGCATTTCAGTCAGTTGCTCAA, reverse – TTGGCCGATTCATTAATGCA.

### Laser microirradiation assay.

For SIRT2 knockdown experiments, U2OS cells were transfected with either nontargeting siRNA or SIRT2 siRNA using RNAiMAX according to the manufacturer’s protocol. After 24 hours after transfection, the medium was replaced with fresh medium, and at 72 hours after transfection, cells were plated onto 35 mm glass-bottomed dishes (MatTek). Laser microirradiation was performed using a Zeiss Observer Z1 microscope equipped with a Micropoint Laser Illumination and Ablation System (Photonic Instruments), using a UV laser with a wavelength of 365 nm. Images were captured every 30 seconds for 10 minutes after damage induction, or cells were fixed 2 minutes after damage induction for subsequent IF assays. For the IF assays, samples were fixed in 4% paraformaldehyde and stained for γH2AX and DAPI after 2 minutes of irradiation.

### Cell survival assay.

U2OS cells were transfected with nonsilencing siRNA or siRNA targeting MRE11, followed by transfection with MRE11-K393Q or MRE11-K393R plasmids, where required. Cells were plated at low density and cultured for 15 days, after which colonies on the plates were fixed and stained with crystal violet. Colony numbers were quantified to assess the impact of MRE11 acetylation on colony survival.

### Biochemical fractionation.

Cytoplasmic and nuclear protein extracts were prepared from HCT116 WT cells, using a nuclear extract kit (Active Motif, 40010) according to the manufacturer’s instructions. Cytoplasmic and nuclear fractions were subjected to IP with an anti-MRE11 antibody. IP products and corresponding input samples were analyzed by SDS-PAGE using antibodies against acetylated Lys (Santa Cruz Biotechnology, sc-8649), lamin A/C (a nuclear marker) (Cell Signaling Technology, 2032T), MRE11 (GeneTex, GTX70212), and α-tubulin (a cytoplasmic marker) (MilliporeSigma, T6074) to assess fractionation purity and MRE11 acetylation status.

### Liquid chromatography coupled to tandem MS.

Immunoprecipitated SFB-MRE11 samples from HCT116 cells after enrichment with anti–FLAG-M2 agarose beads were reduced with 5 mM DTT for 15 minutes at 37°C and then alkylated with 20 mM iodoacetamide for 30 minutes at 37°C. The samples were resolved on a 10% polyacrylamide SDS gel and after staining with Coomassie G-250, the proteins were excised and subjected to in-gel digestion (12.5 ng/μL trypsin) overnight at 37°C. Extracted peptides were loaded onto a C18 column, eluted and detected by Orbitrap (300–1,600 mass per charge ratio [*m/z*], 1,000,000 automatic gating control [AGC] target, 1,000 ms maximum ion time, resolution 30,000). Tandem MS (MS/MS) scans in a linear trap quadrupole MS (2 *m/z* isolation width, 35% collision energy, 5,000 AGC target, 150 ms maximum ion time; Thermo Finnigan, Thermo Fisher Scientific) were acquired by data-dependent acquisition. All data were converted from raw files to the.dta format using ExtractMS, version 2.0 (ThermoElectron), and searched against a human reference database downloaded from the National Center for Biotechnology Information using the SEQUEST Sorcerer algorithm (version 3.11; SAGE-N Research). Searching parameters included mass tolerance of precursor ions (±50 ppm) and product ion (±0.5 *m/z*), fully tryptic restriction, with a dynamic mass shift for oxidized methionine (+15.9949) and acetylated Lys (+42.0106), 4 maximal modification sites, and a maximum of 2 missed cleavages. Only b and y ions were considered during the database match.

To evaluate the FDR, all original protein sequences were reversed to generate a decoy database that was concatenated to the original database. The FDR was estimated by the number of decoy matches (nd) and total number of target matches (nt), as follows: FDR = 2 × nd/nt, assuming mismatches in the original database were the same as in the decoy database. To remove false-positive matches, assigned peptides were grouped by a precursor ion charge state, and each group was first filtered by mass accuracy (10 ppm for high-resolution MS) and by dynamically increasing the correlation coefficient (Xcorr) and ΔCn values to reduce the protein FDR to less than 1%. Peptide abundance was based on peptide extracted ion intensity as previously reported (90). Accurate peptide mass (±10 ppm) and retention time was used to derive signal intensity for each peptide across liquid chromatography–MS/MS runs.

### Multisequence alignment.

The MRE11 protein sequences from various organisms were aligned using the Clustal Omega program, and the alignment was further processed in the BoxShade program to highlight conserved amino acids.

### Plasmids.

The following plasmids were used: SFB-MRE11 WT; SFB-MRE11 K393Q; SFB-MRE11 K393R; GFP-MRE11; GFP-MRE11 K393Q; and GFP-MRE11 K393R. SFB-MRE11 WT was a gift from Junjie Chen (The University of Texas MD Anderson Cancer Center, Houston, Texas, USA). All other MRE11-expressing plasmids were made with the pcDNA3.1 backbone by the Emory Integrated Genomics Core. SIRT2-FLAG WT and H187Y were a gift from David Gius (University of Texas Health Science Center at San Antonio, San Antonio, Texas, USA).

### Generation of CRISPR/cas9 SIRT2 KO cells.

HCT116 cells were transfected with a plasmid containing a Cas9-GFP construct along with guide RNAs targeting SIRT2 exon 6 (5′-CGGGCTCAAGTTCCGCTTCGGG-3′) or a nontargeting control. After 72 hours after transfection, cells were harvested and sorted based on GFP expression using FACS. Subsequently, Western blot analysis was performed to test for KO of SIRT2 in the resulting cell lines.

### Statistics.

The statistical details of each experiment can be found in the figure legends. The number of experimental replicates is indicated in the figure legends or outlined in Methods. Statistical comparisons included 1- and 2-way ANOVA or 2-tailed Student’s *t* test, as specified in the figure legends, using GraphPad Prism software, version 7.04 (GraphPad Software) or Excel (Microsoft). Statistical significance was set at *P* < 0.05. Data presented in the graphs represent the mean ± SD of 3 replicates, unless stated otherwise.

### Data availability.

Supporting data are provided in a Supporting Data Values file or otherwise is available from the corresponding author upon request.

## Author contributions

FS, HZ, and DSY conceived and designed the study. The order of co-first authors was determined as follows: FS is listed first as first author because she took over the project from HZ, completed the writing, and contributed to revisions for the manuscript. HZ is listed second because she initiated the study and found the initial observation. FS, HZ, PKV, ATJ, MEE, AJB, NCL, XL, PEH, and DMD performed the experiments. FS, HZ, PKV, ATJ, MEE, AJB, NCL, XL, PEH, DMD, XY, ZSB, XD, NTS, and DSY interpreted the experiments. FS and DSY wrote the manuscript with input from all authors.

## Funding support

This work is the result of National Institutes of Health (NIH) funding, in whole or in part, and is subject to the NIH Public Access Policy. Through acceptance of this federal funding, the NIH has been given a right to make the work publicly available in PubMed Central.

- NIH/National Cancer Institute (NCI) (grants R01CA178999, R01CA254403, R01CA254403-S1, R01CA254403-S2, R01CA301614, and U54CA274513, to DSY).
- NIH/National Institutes of General Medical Sciences (grant 5K12GM000680 IRACDA FIRST to FS and R35GM155032 to XL).
- The Emory Integrated Genomics Core Shared Resource of Winship Cancer Institute of Emory University, Emory Integrated Proteomics Core, Flow Cytometry Core, and NIH/NCI (grant P30CA138292).
- Winship Cancer Institute Brenda Nease Pilot Award.

## Supplementary Material

Supplemental data

Unedited blot and gel images

Supporting data values

## Version 1 01/08/2026

In-Press Preview

## Version 2 03/02/2026

Electronic publication
