# Supplementary material for: MRE11 deacetylation by SIRT2 promotes DNA binding to facilitate DNA end resection and ATM-dependent signaling
Source: J Clin Invest. 2026 Jan 8;136(5):e186711. doi: 10.1172/JCI186711 (PMC12948433; doi:10.1172/JCI186711)
Supplement: Supplemental data [file jci-136-186711-s154.pdf]

SUPPLEMENTAL INFORMATION

Figure S1. SIRT2 deacetylation promotes DNA end resection. U2OS cells were transfected with SIRT2 or NT siRNA, and GFP-RPA70, then subjected to laser microirradiation. Representative image shows analysis of RPA accumulation at sites of laser-induced DNA damage 2 min after laser microirradiation. The experiment was conducted 3 independent times.

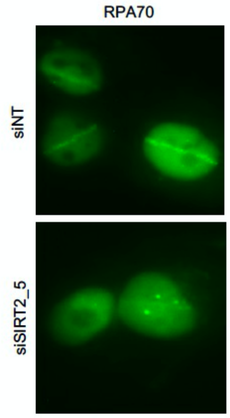

Supplemental Figure S1

Figure S2. SIRT2 depletion impairs DNA end resection as measured by BrdU ssDNA formation. **(A-C)** HeLa cells depleted for SIRT2, CtIP or 53BP1 were treated with 30 μM BrdU for 36 h followed by further 4h incubation with 6 Gy IR. Cells were fixed under non-denaturing conditions and processed for IF using anti-BrdU (ssDNA) and γH2AX antibodies. **(A)** Representative images and **(B)** quantitation of BrdU foci intensity among γH2AX positive cells are shown. Mean +/- SD are shown for 3 independent experiments, each consisting of 25 cells. **(C)** Western blot analysis showing confirmation of SIRT2, CtIP and 53BP1 depletion in HeLa used in A-B. Western blots shown are representative and each experiment was conducted at least 3 independent times. Statistical significance was determined using one-way ANOVA followed by Dunnett's post-hoc test for multiple comparisons. **** $p < 0.0001$.

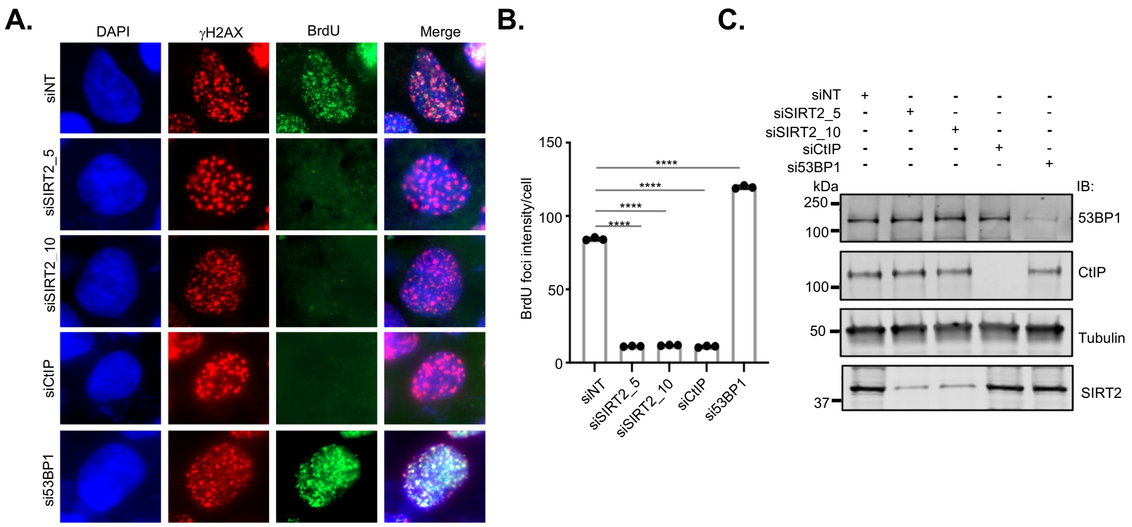

Supplemental Figure 2

Figure S3. MRE11 is acetylated at K393 and impairs binding to DSBs. (A) LC-MS/MS analysis showing site specific acetylation of MRE11 at K393, K416, and K673. **(B)** Impact prediction of acetylation at K393, K416, and K673 using SIFT analysis and PolyPhen-2 analysis. **(C)** Biochemical fractionation of MRE11 in HCT116 cells showing deacetylation of nuclear MRE11 4h after 6 Gy IR. A representative image is shown. **(D)** Radio-resistant (IRR) - A549 cells were transfected with GFP-MRE11 plasmids, exposed to 6 Gy IR where indicated and recovered for 4h. Lysates were incubated in binding buffer with and without biotin-dsDNA oligos and the biotinylated oligos were pulled down with agarose-streptavidin beads. Proteins associated with biotin dsDNA were western blotted with GFP antibody. Input DNA was included and probed with GFP and Tubulin (loading control) antibodies. Shown is a representative image. **(E-F)** Quantitative analysis of MRE11-binding to FokI-induced DSBs using ChIP-qPCR, done three independent times. Mean +/- SD is shown. *** $p < 0.001$, ns = not significant using a student's t-test. **(G)** HCT116 or HCT116 cells expressing GFP-FLAG-MRE11 WT, K393Q, or K393R were treated with camptothecin (CPT). Cells were harvested and lysates were co-IP'ed with anti-FlAG antibody. The samples were run on SDS-PAGE and probed with indicated antibodies. A representative image is shown. Western blots shown are representative and each experiment was conducted at least 3 independent times. Statistical significance was determined using one-way ANOVA followed by Dunnett's post-hoc test for multiple comparisons. *** $p < 0.001$, **** $p < 0.0001$, and ns = not significant.

A.

POS	SEQ	3LATOM	SCORE (Normalized)	CONFIDENCE INTERVAL COLORS	MSA DATA	RESIDUE VARIETY
393	K	LYS393:A	-0.478	7,6	148/150	N,C,R,K,T,S,I,M,E,A,Q
416	K	LYS416:A	2.405	1,1	124/150	K,R,P,L,N,Q,A,E,M,V,F,G,S,T,D
673	K	LYS673:A	0.826	4,1	87/150	Q,V,S,T,I,K,R,N,X

B.

Site	SIFT Analysis	PolyPhen-2 (out of 1)
K393	K->Q or R predicted to affect protein function	0.999 predicted to be damaging
K416	K-> Q or R tolerated	0.004 predicted to be benign
K673	K->Q predicted to affect protein function (low confidence) K->R tolerated	0.007 predicted to be benign

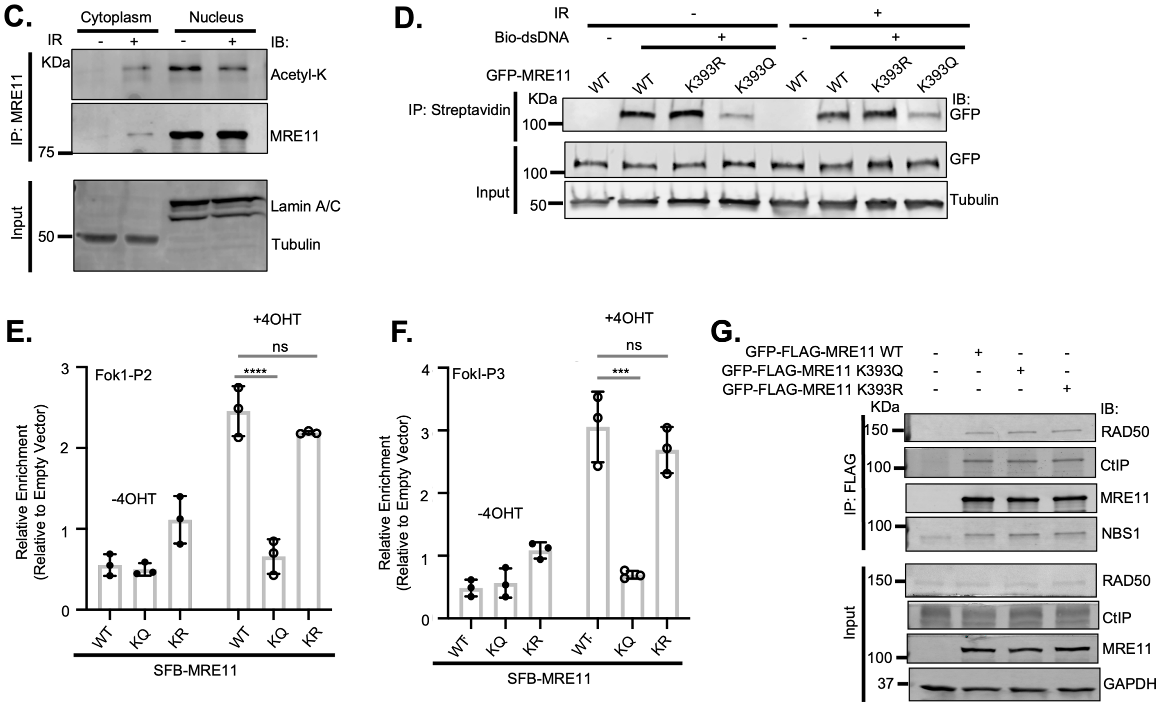

Supplemental Figure S3

Figure S4. MRE11 K393 acetylation does not impact phosphorylation of NBS1 at S343. (A) HCT116 WT or *SIRT2* KO cells were exposed to 6 Gy IR and allowed to recover for 4h. Cells were processed for SDS-PAGE followed by western analysis with the indicated antibodies. **(B)** IRR– A549 cells were transfected with non-silencing or MRE11 siRNA and then further transfected with GFP-FLAG-MRE11 plasmids, where indicated. Cells were treated with 6 Gy IR, recovered for 4h and then processed for SDS-PAGE and western analysis with the indicated antibodies. Western blots shown are representative and each experiment was conducted at least 3 independent times.

A.

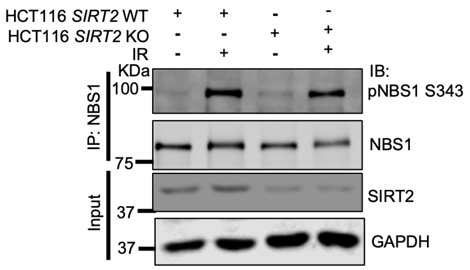

B.

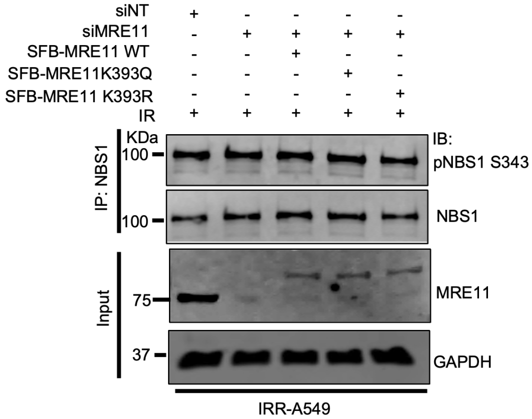

Supplemental Figure S4
