# Supplementary material for: MRE11 deacetylation by SIRT2 promotes DNA binding to facilitate DNA end resection and ATM-dependent signaling
Source: J Clin Invest. 2026 Jan 8;136(5):e186711. doi: 10.1172/JCI186711 (PMC12948433; doi:10.1172/JCI186711)

Fully unedited blot for Figure 1A

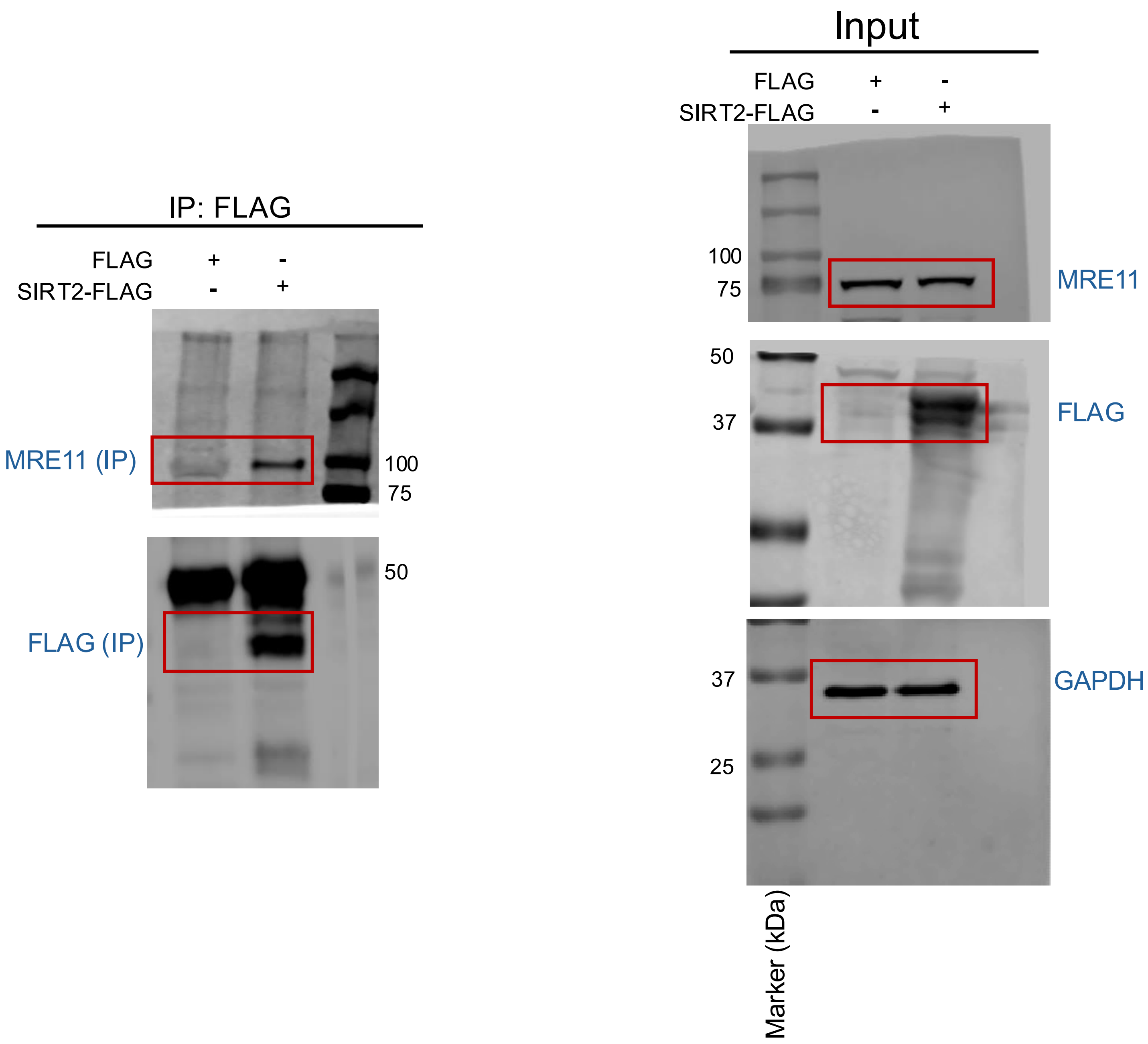

Fully unedited blot for Figure 1B

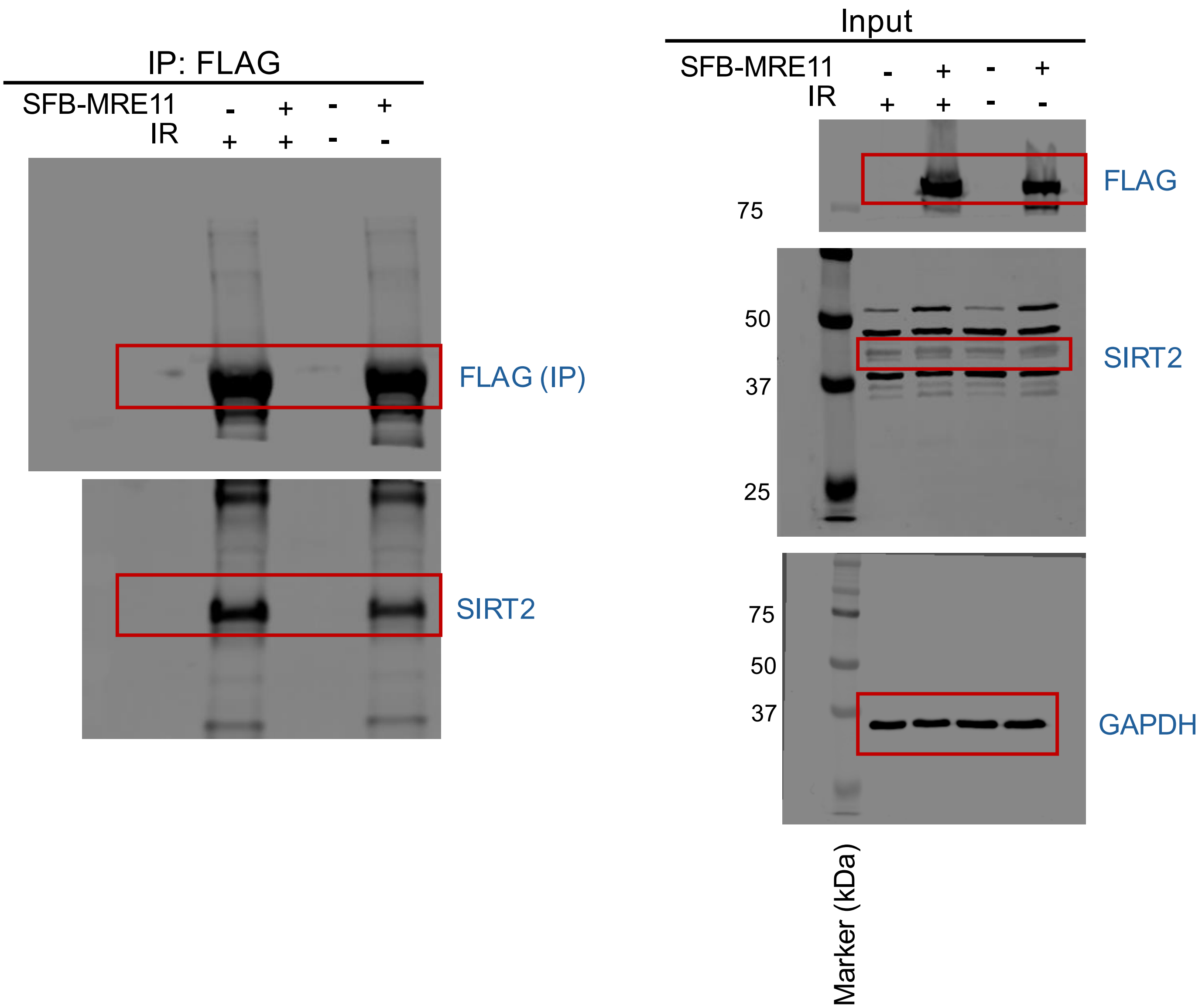

Fully unedited blot for Figure 1C

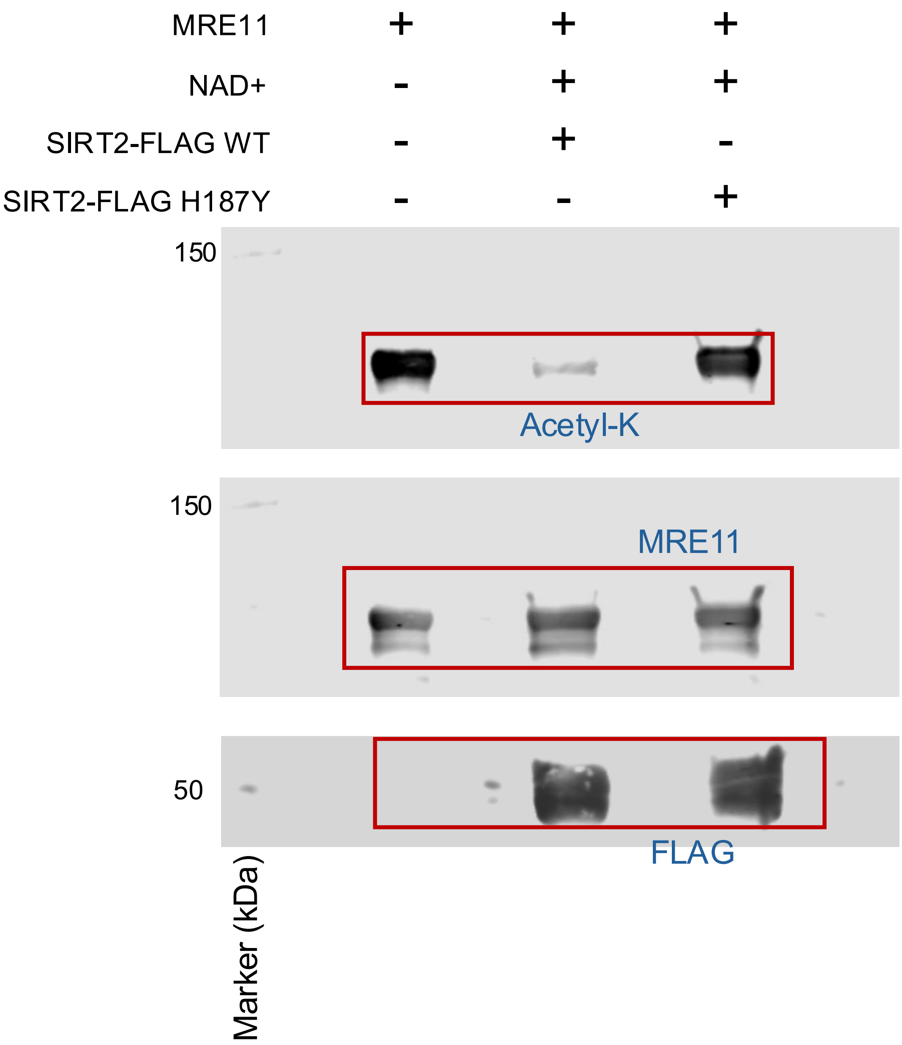

Fully unedited blot for Figure 1D

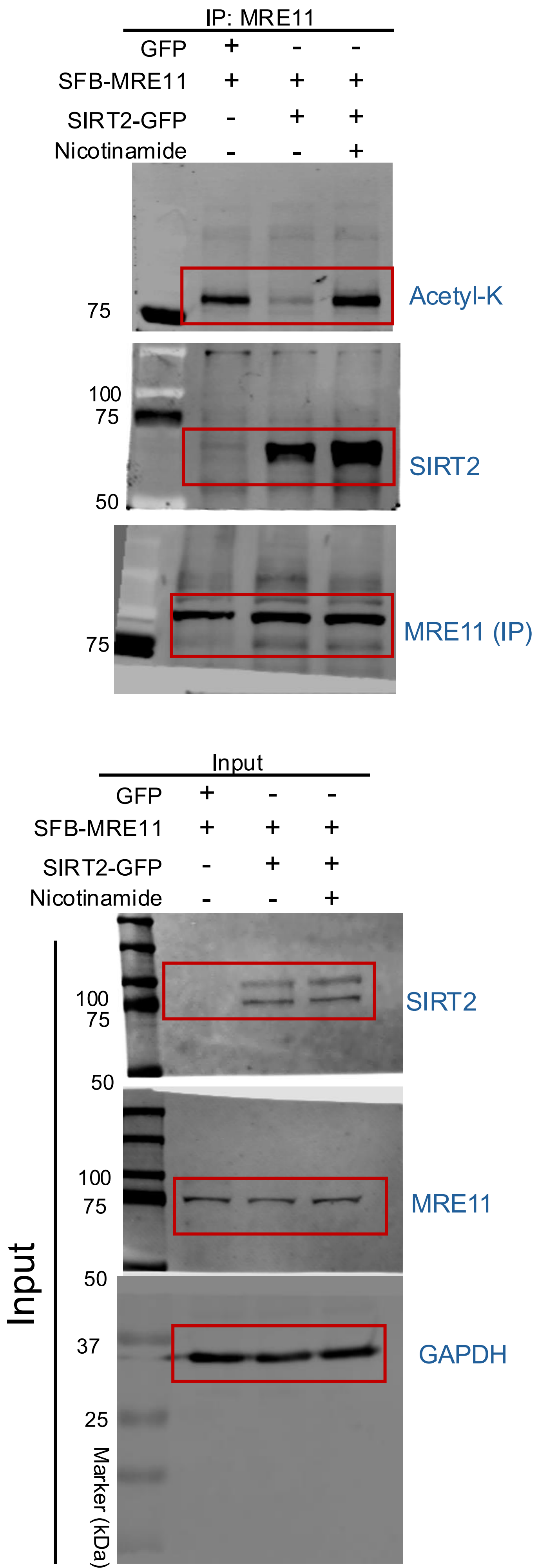

Fully unedited blot for Figure 2C

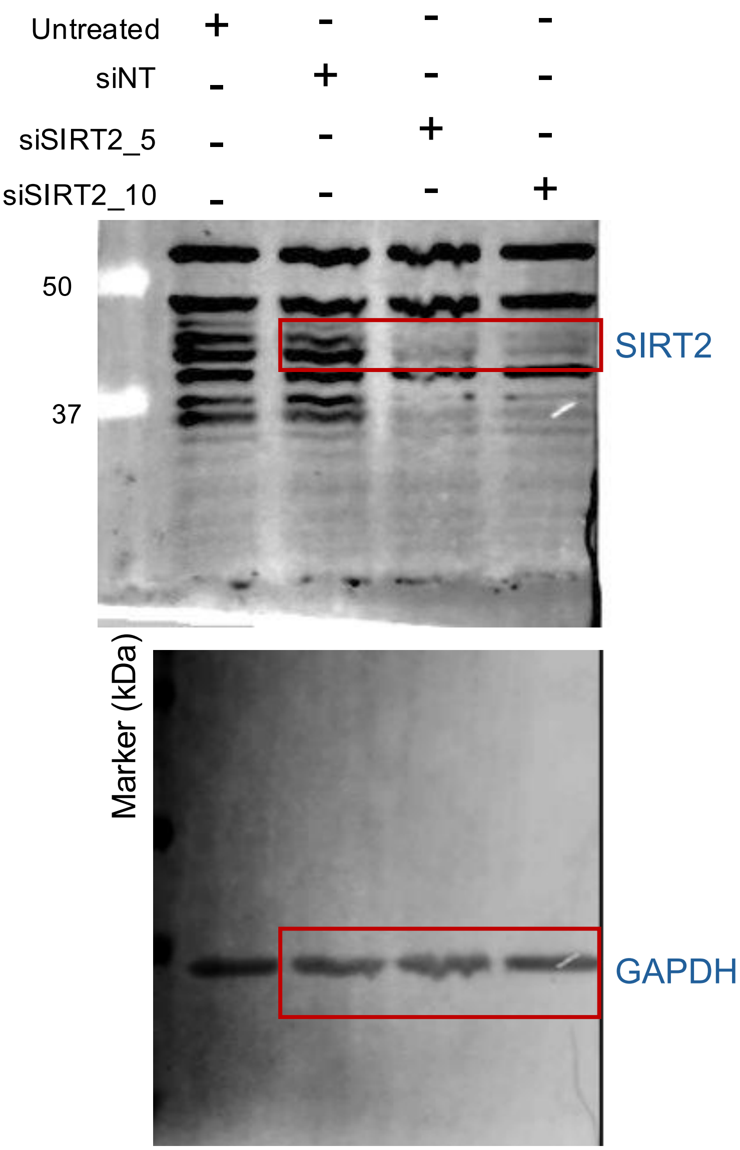

Fully unedited blot for Figure 3C

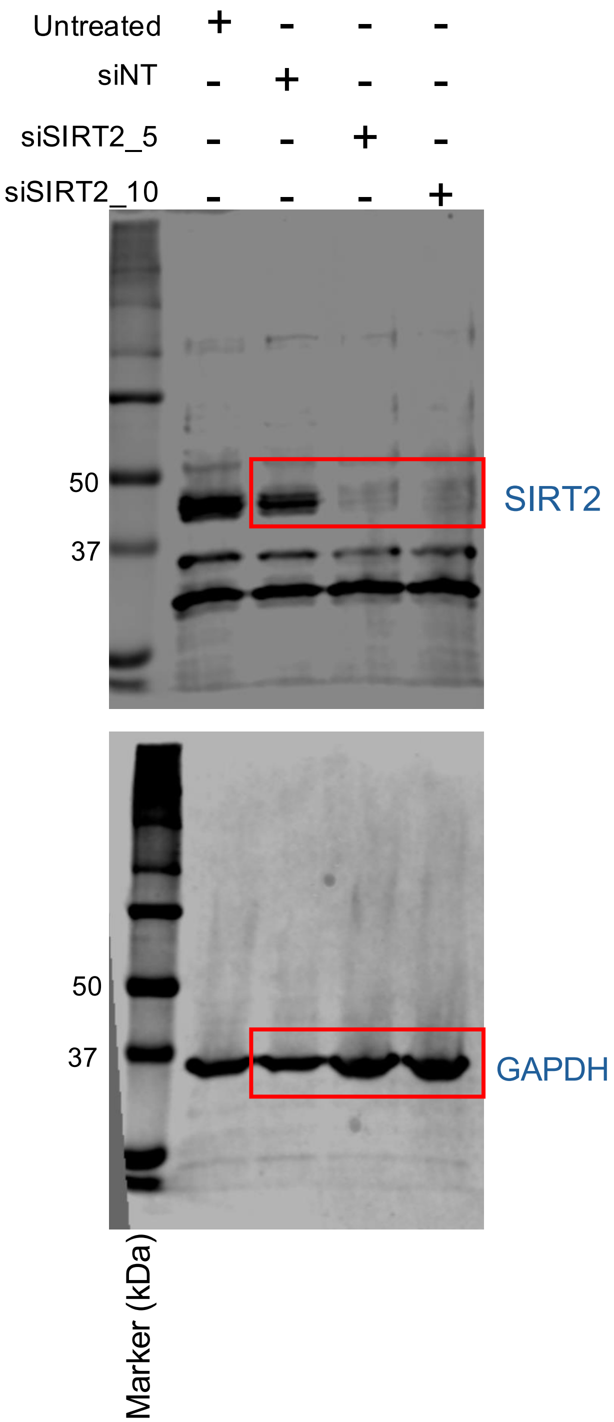

Fully unedited blot for Figure 3F

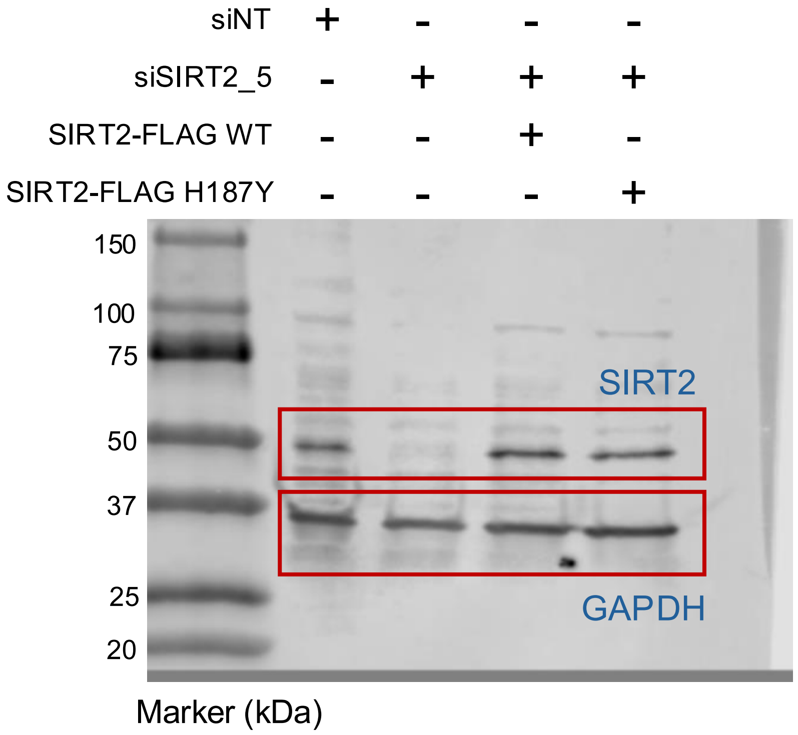

Fully unedited blot for Figure 4B

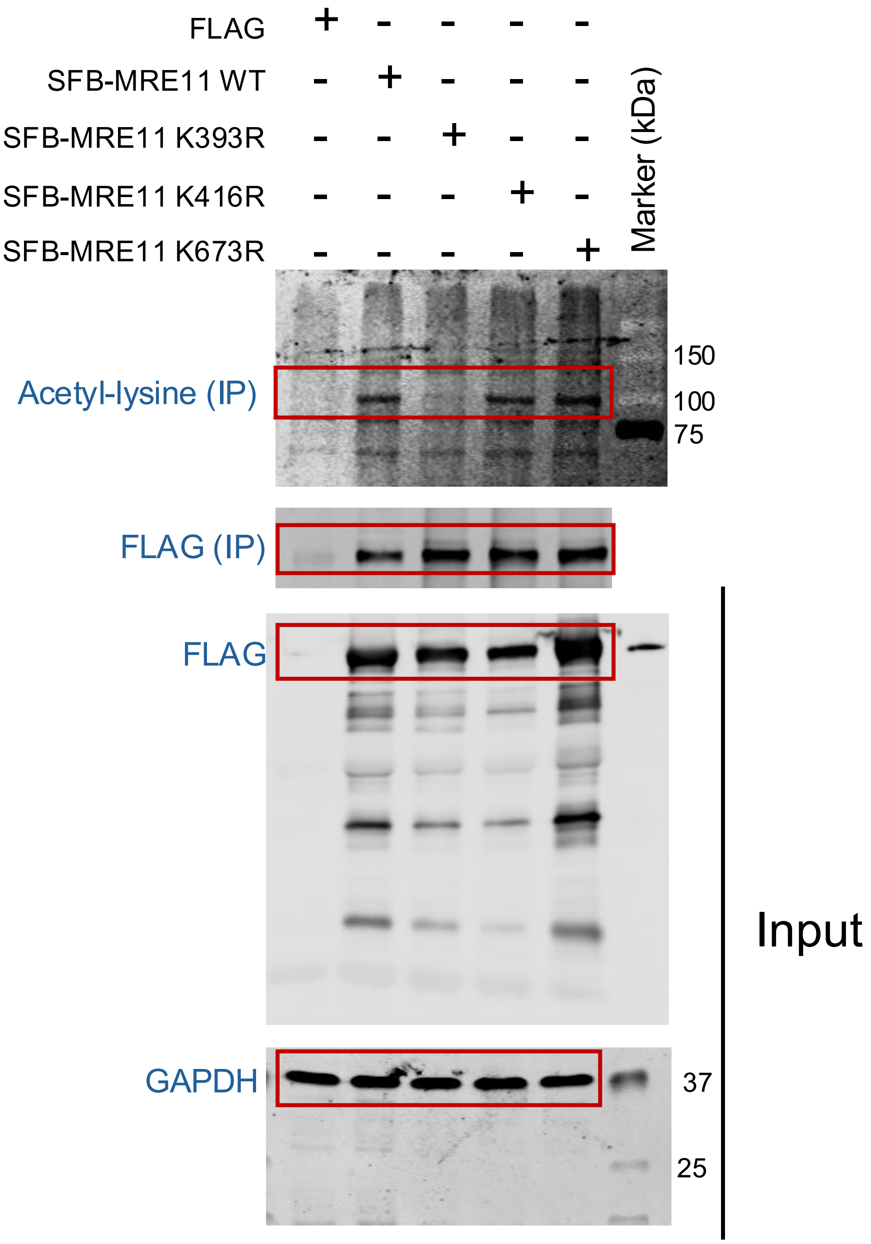

Fully unedited blot for Figure 4C

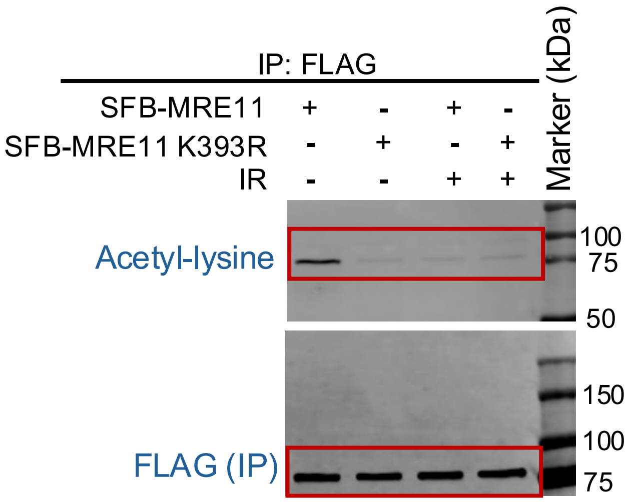

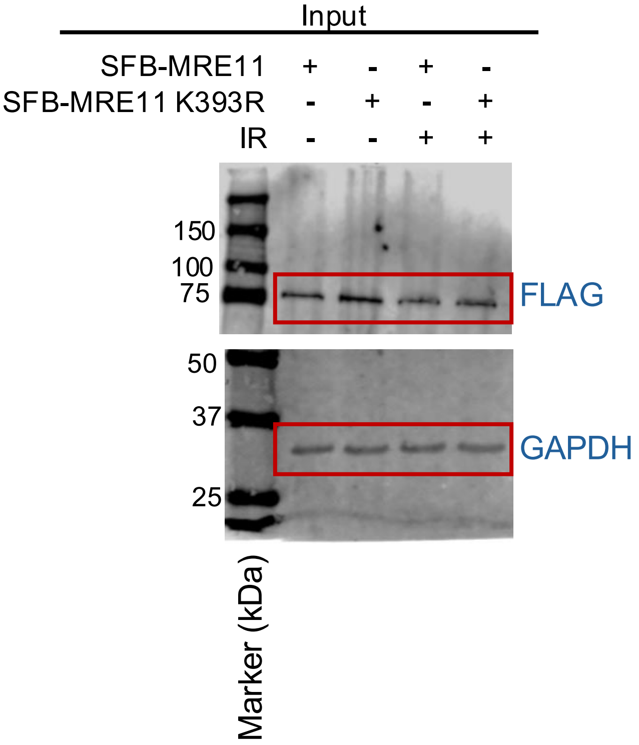

Fully unedited blot for Figure 5B

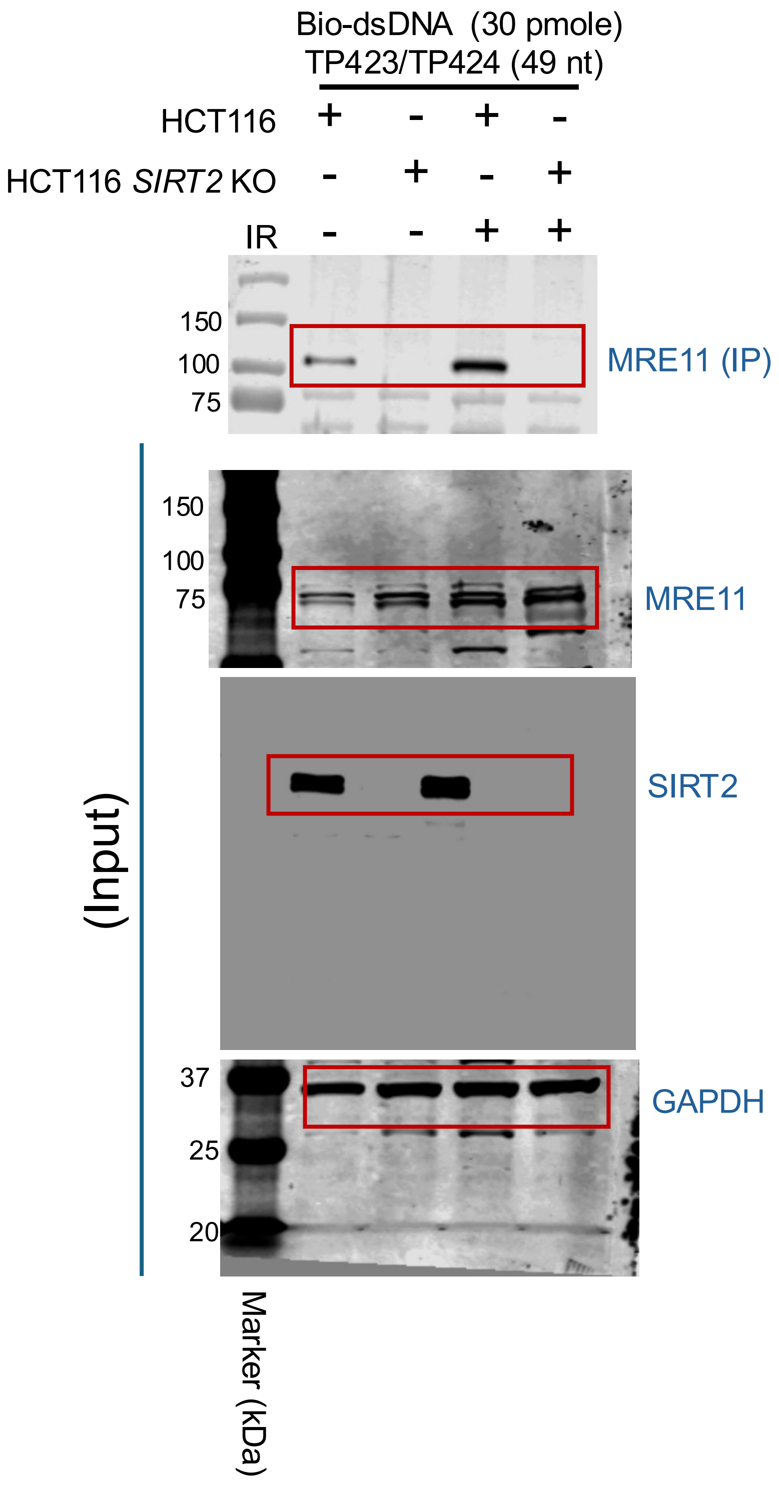

Fully unedited blot for Figure 5C

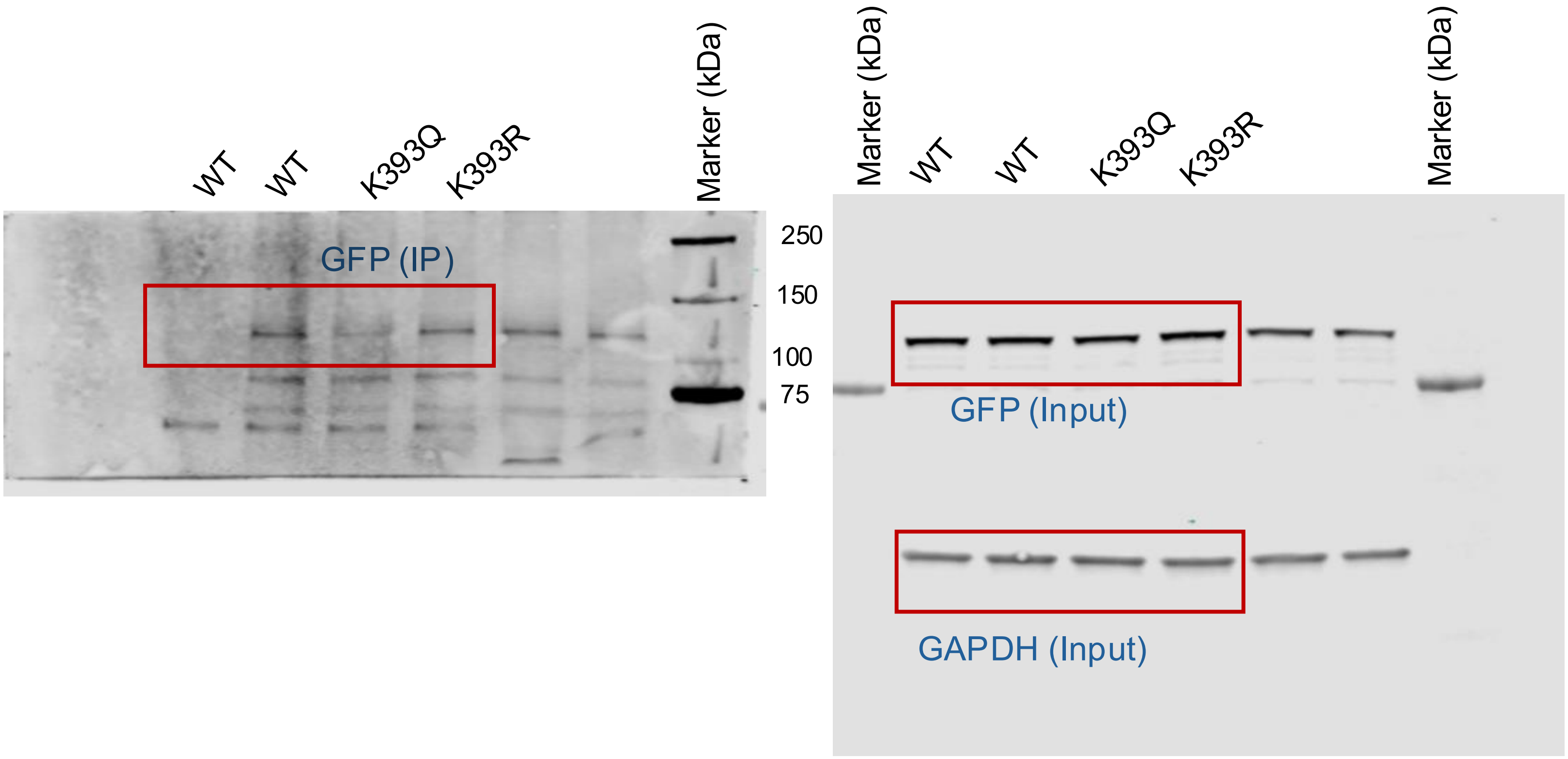

Fully unedited blot for Figure 6C

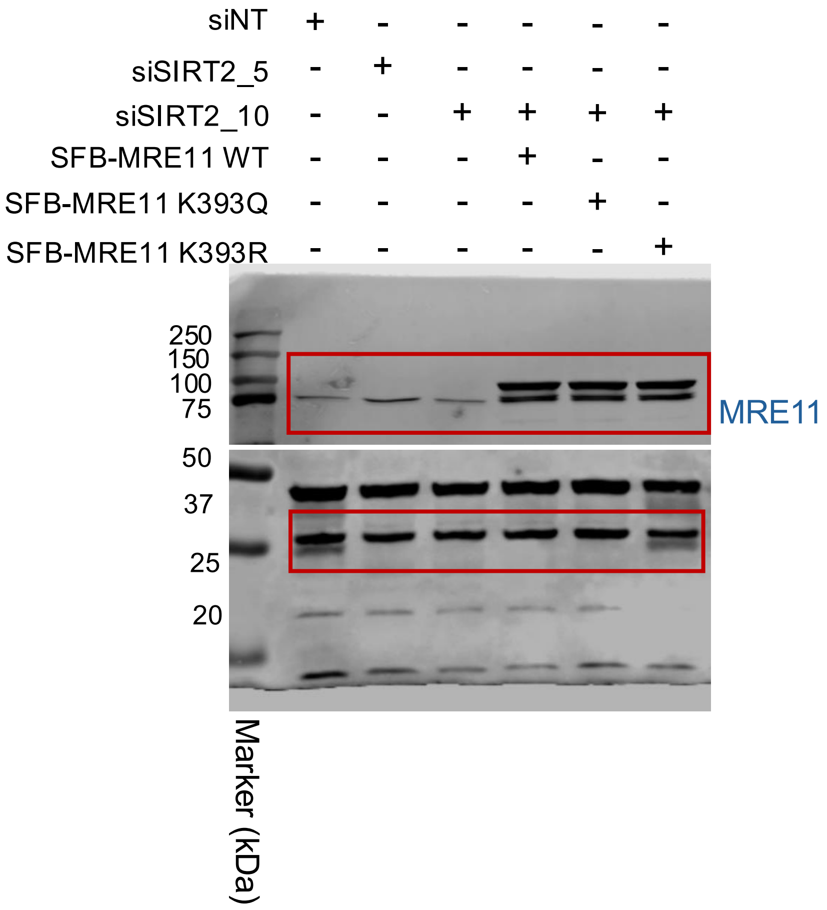

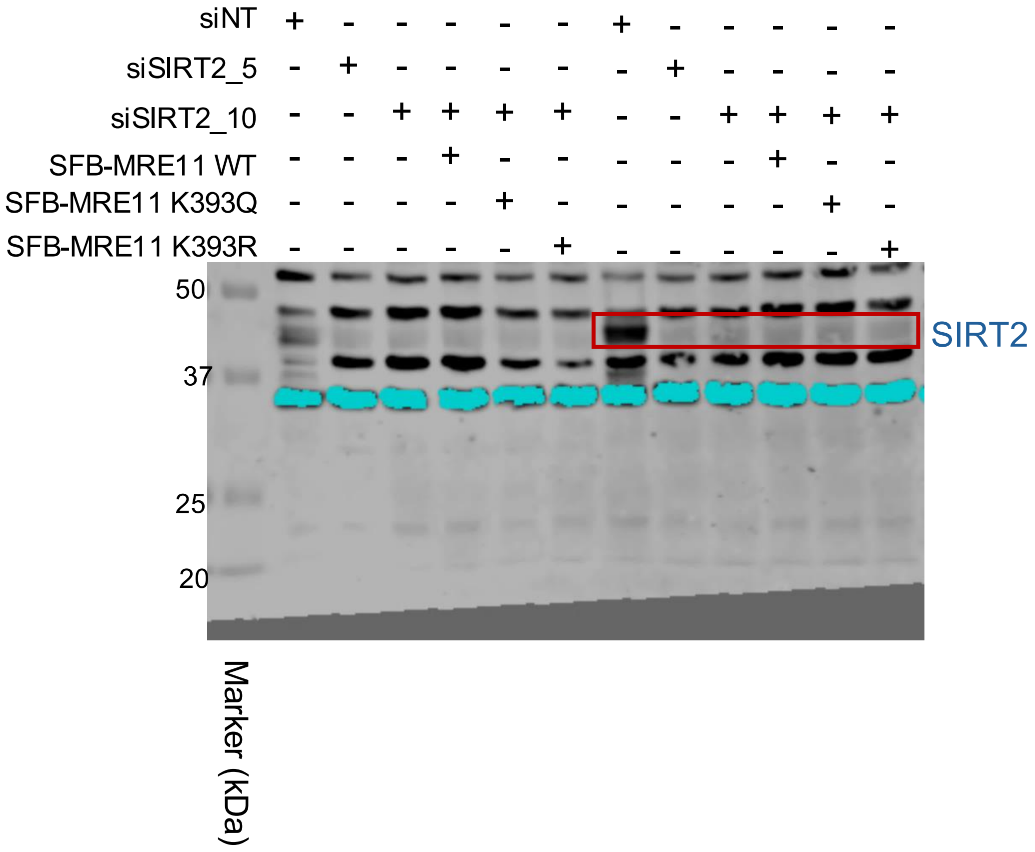

Fully unedited blot for Figure 7C

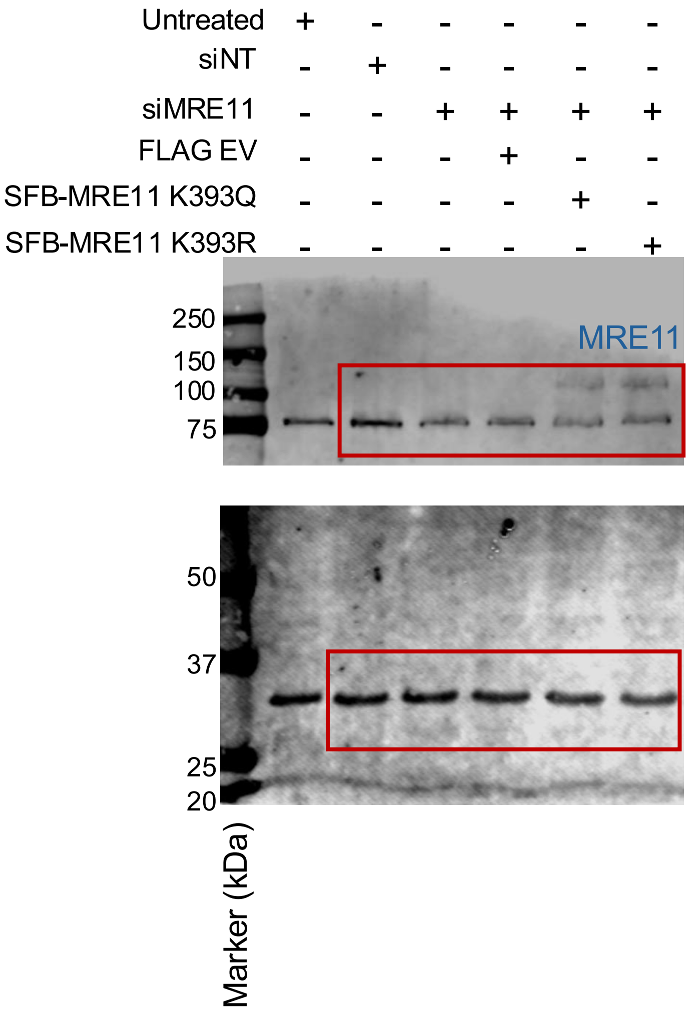

Fully unedited blot for Figure 8A

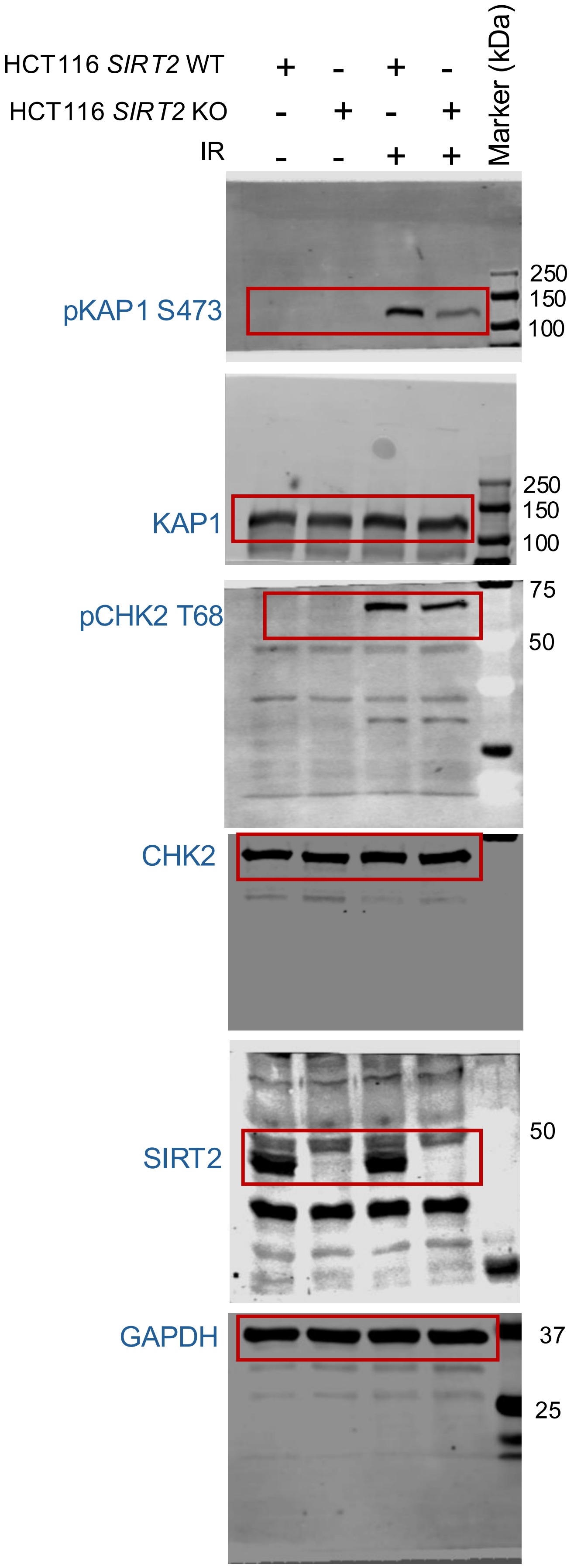

Fully unedited blot for Figure 8C

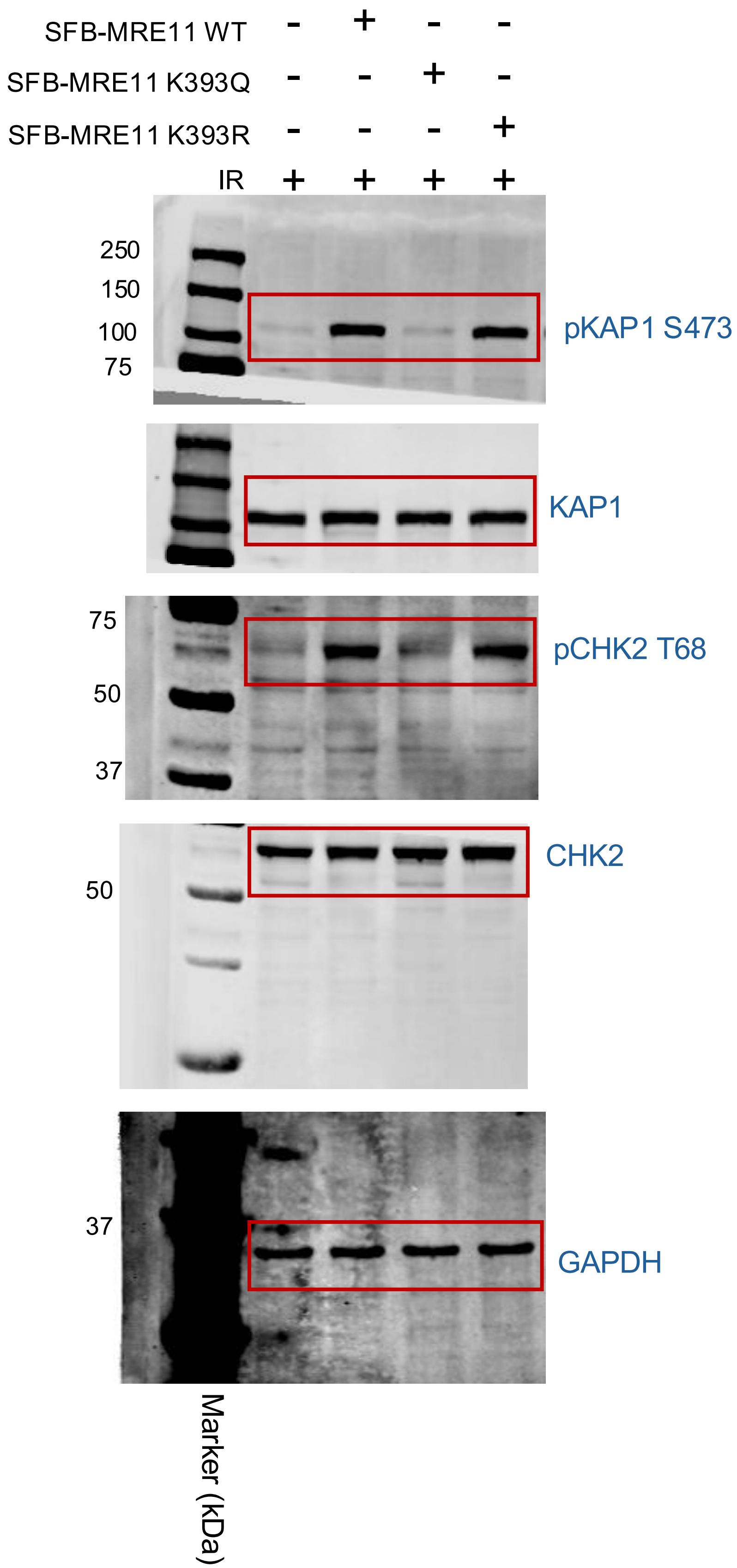

Fully unedited blot for Figure S2C

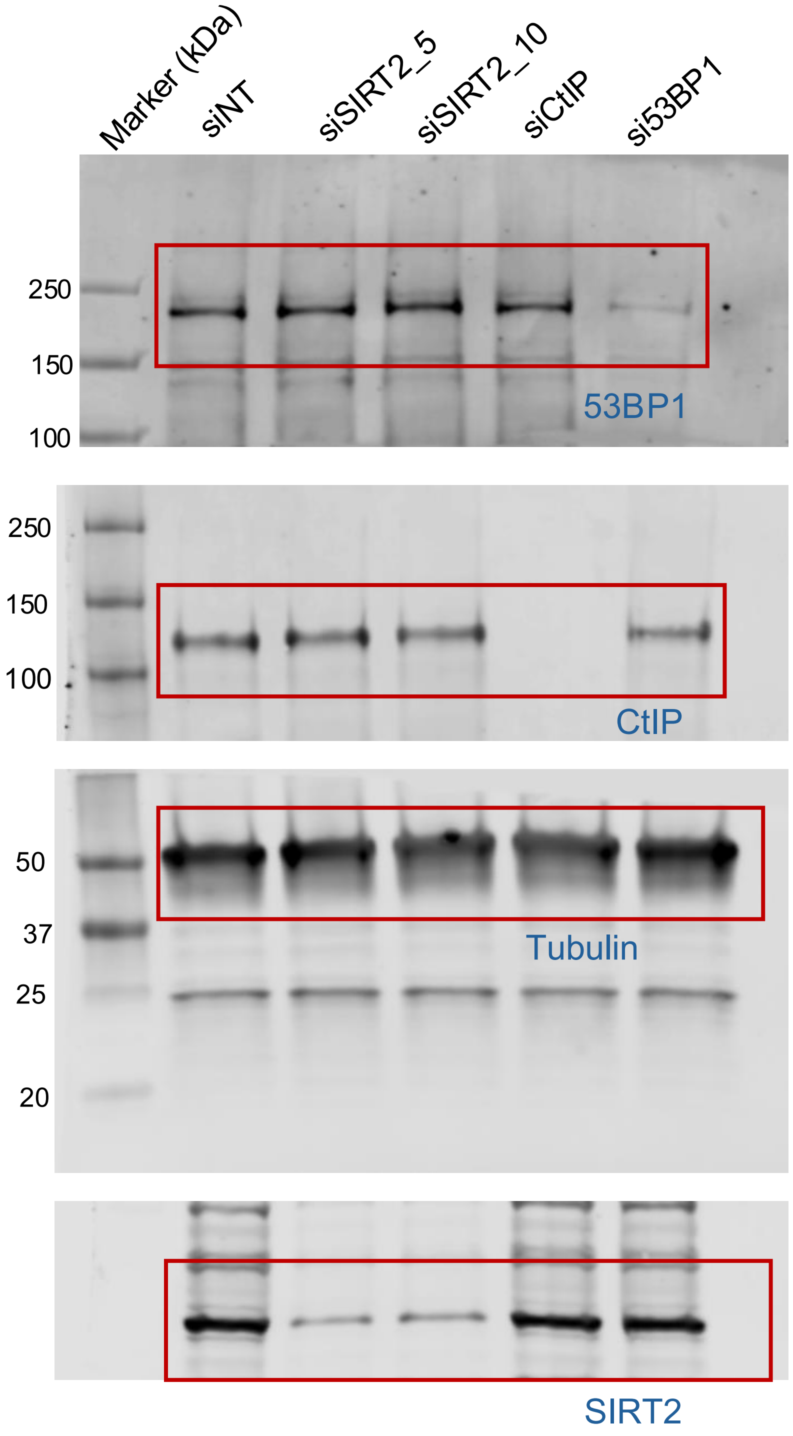

Fully unedited blot for Supplemental Figure S3C

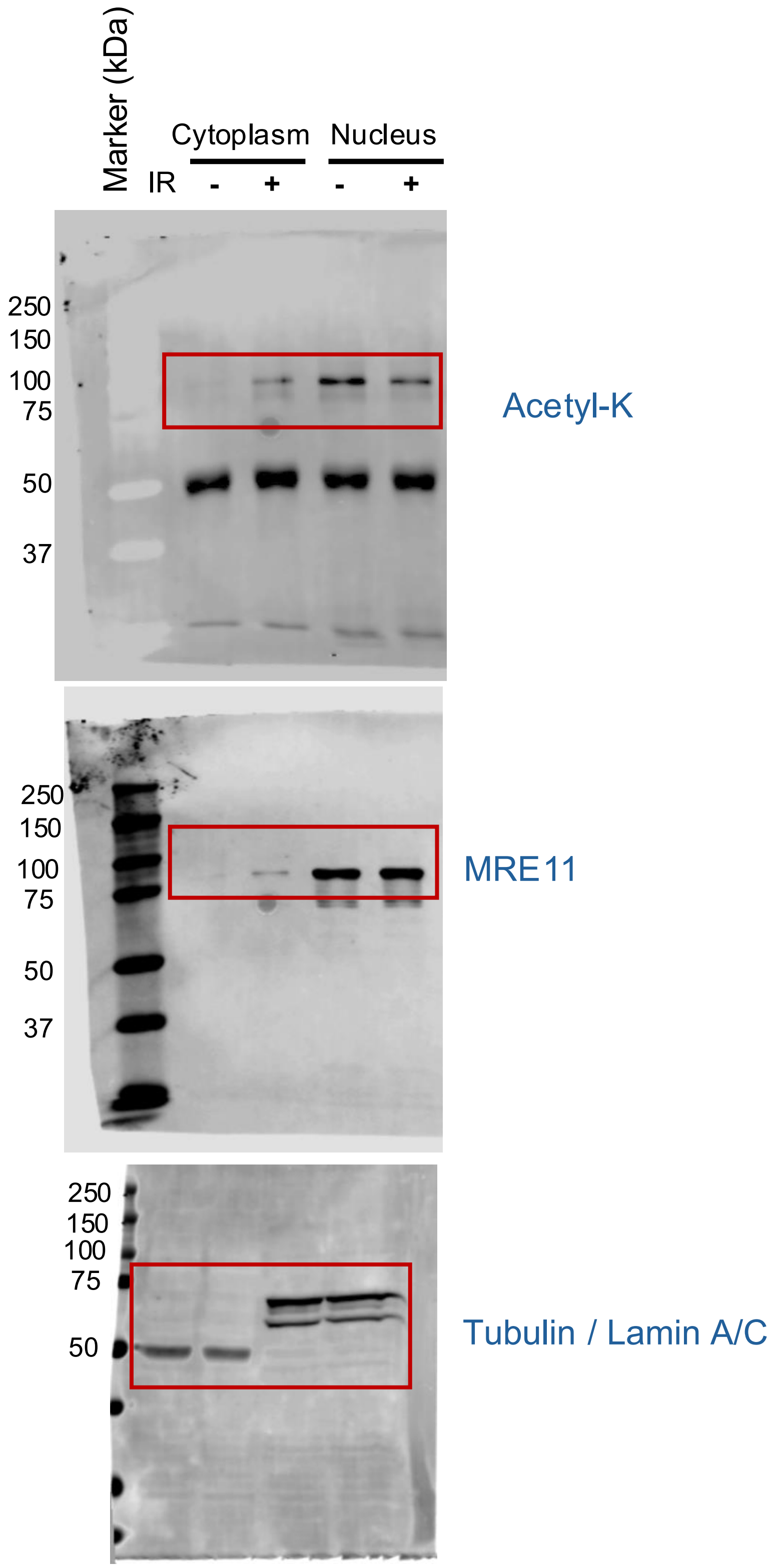

Fully unedited blot for Supplemental Figure S3D

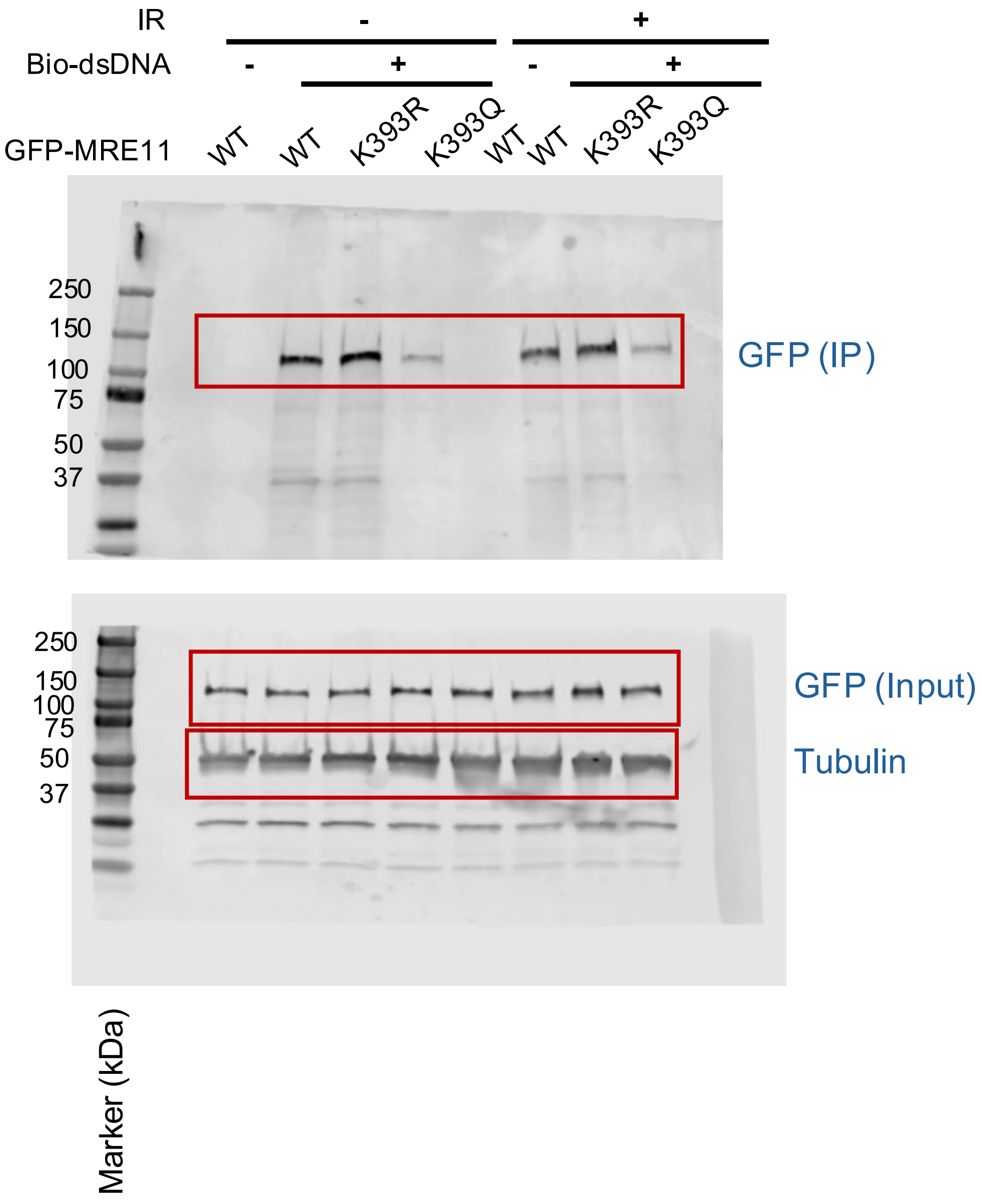

Fully unedited blot for Supplemental Figure S3G

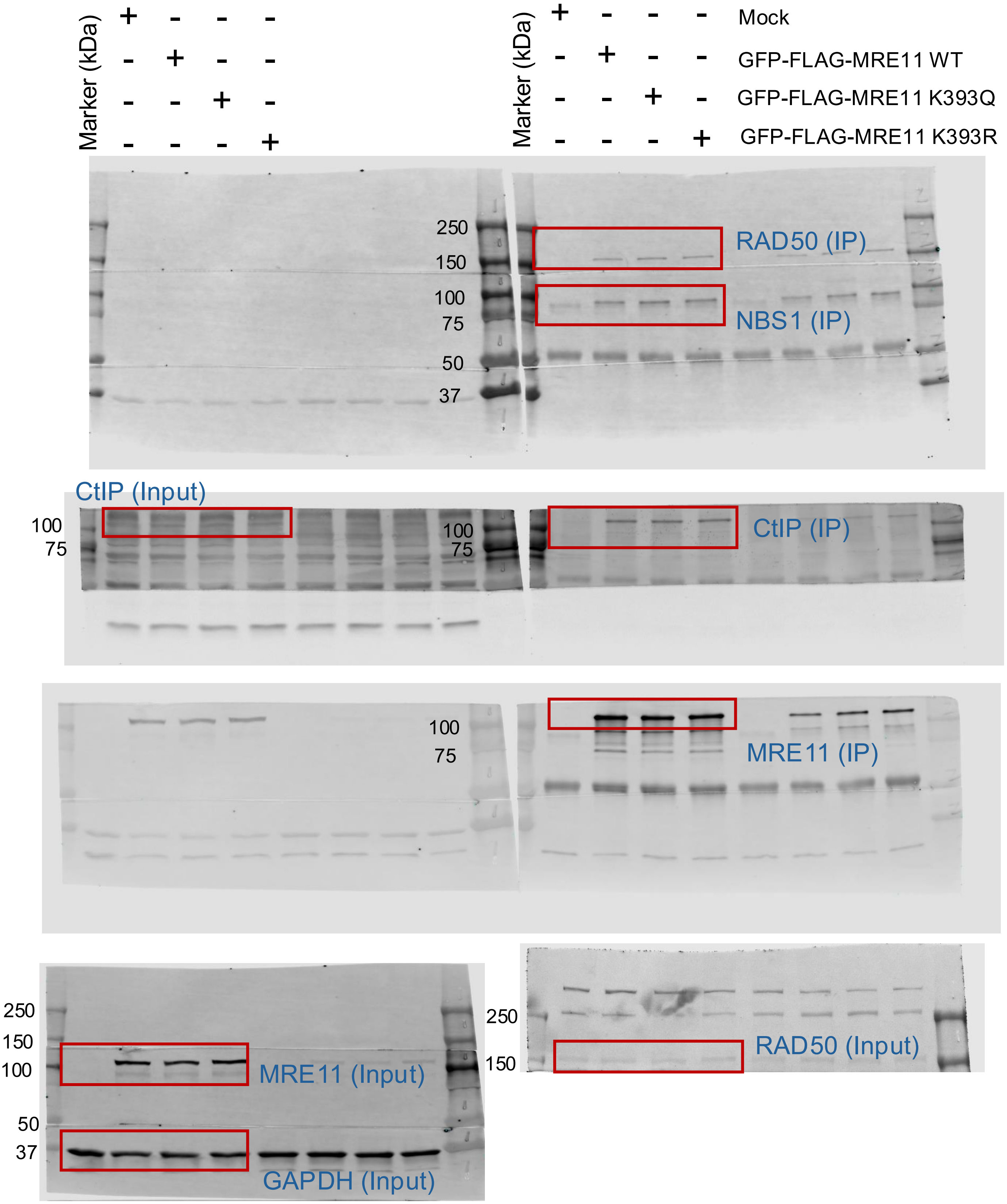

Fully unedited blot for Supplemental Figure S4A

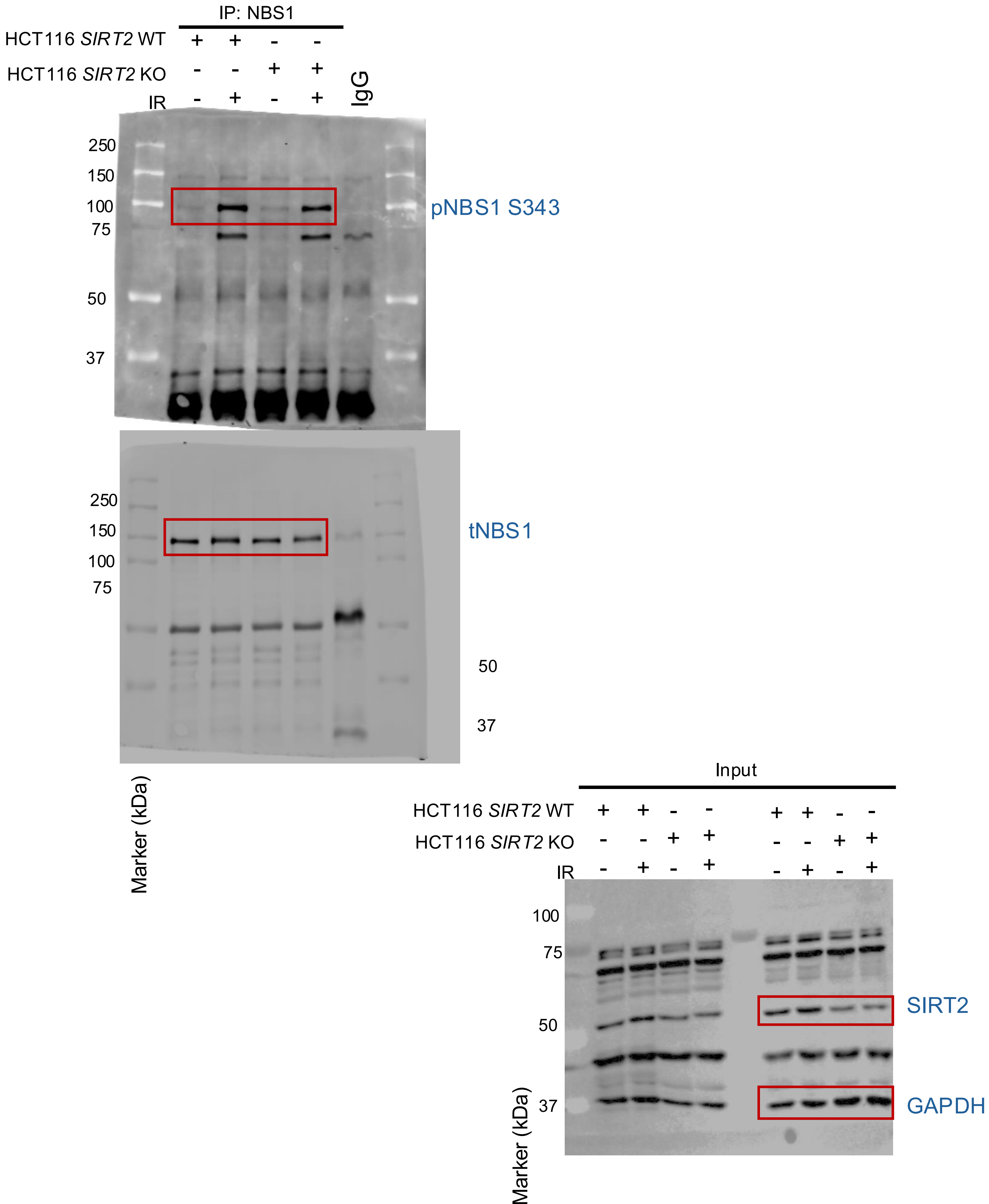

Fully unedited blot for Supplemental Figure S4B

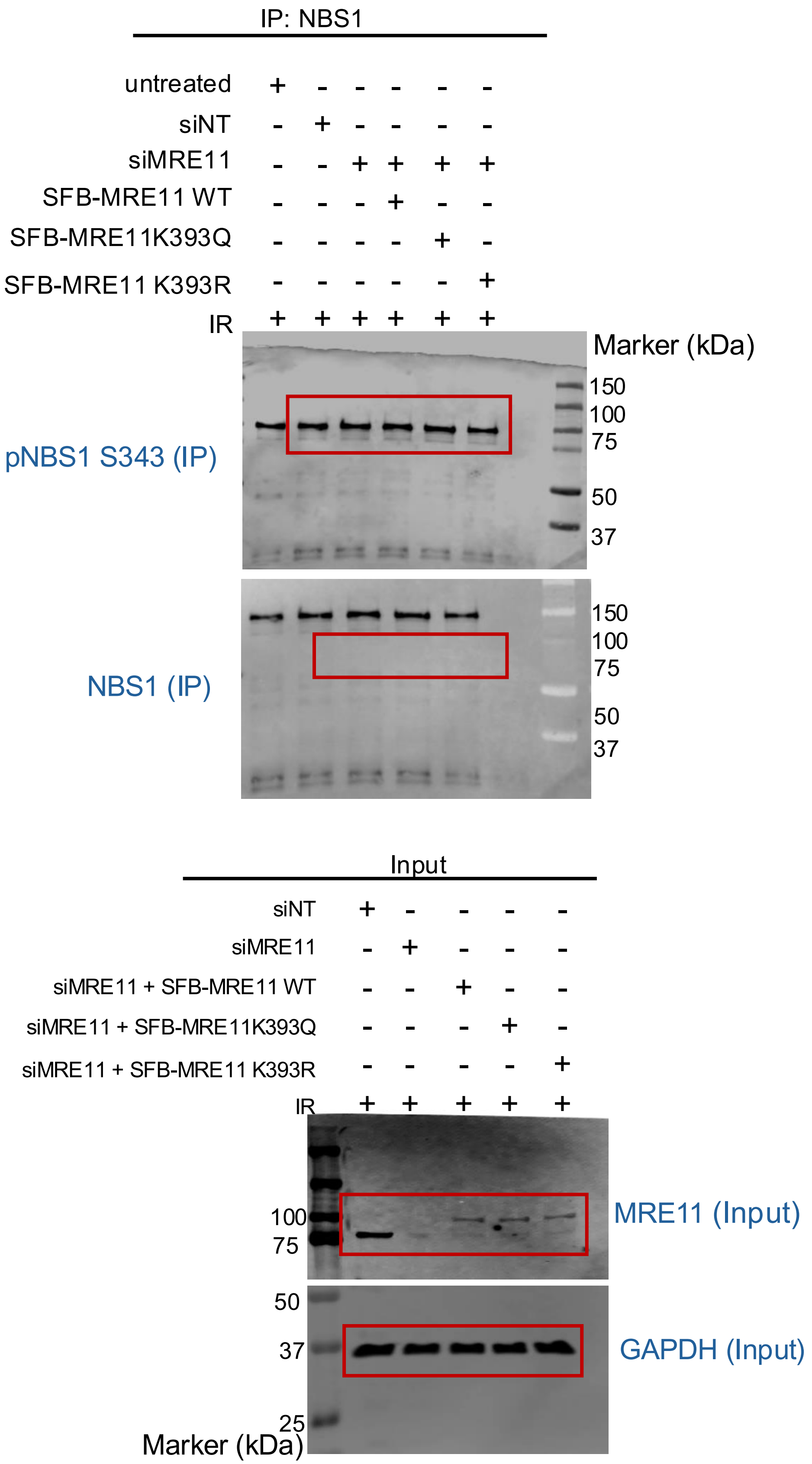

Supplement: Unedited blot and gel images [file jci-136-186711-s155.pdf]
